# Highly Significant Antiviral Activity of HIV-1 LTR-Specific Tre-Recombinase in Humanized Mice

**DOI:** 10.1371/journal.ppat.1003587

**Published:** 2013-09-26

**Authors:** Ilona Hauber, Helga Hofmann-Sieber, Jan Chemnitz, Danilo Dubrau, Janet Chusainow, Rolf Stucka, Philip Hartjen, Axel Schambach, Patrick Ziegler, Karl Hackmann, Evelin Schröck, Udo Schumacher, Christoph Lindner, Adam Grundhoff, Christopher Baum, Markus G. Manz, Frank Buchholz, Joachim Hauber

**Affiliations:** 1 Heinrich Pette Institute – Leibniz Institute for Experimental Virology, Hamburg, Germany; 2 Department of Medical Systems Biology, University Hospital and Medical Faculty Carl Gustav Carus, Technische Universität Dresden, Dresden, Germany; 3 Friedrich-Baur-Institute, Department of Neurology, Ludwig-Maximilians-University Munich, Munich, Germany; 4 Infectious Diseases Unit, I. Department of Internal Medicine, University Medical Center Hamburg-Eppendorf, Hamburg, Germany; 5 Institute of Experimental Hematology, Hannover Medical School, Hannover, Germany; 6 Division of Hematology/Oncology, Children's Hospital Boston, Harvard Medical School, Boston, Massachusetts, United States of America; 7 Institute for Research in Biomedicine, Bellinzona, Switzerland; 8 Klinik für Onkologie, Hämatologie und Stammzelltransplantation, RWTH Aachen University, Aachen, Germany; 9 Institute for Clinical Genetics, University Hospital and Medical Faculty Carl Gustav Carus, Technische Universität Dresden, Dresden, Germany; 10 Institute for Anatomy and Experimental Morphology, University Cancer Center Hamburg, University Medical Center Hamburg-Eppendorf, Hamburg, Germany; 11 Department of Gynecology, Day Kimball Healthcare Hospital, Hamburg, Germany; 12 University and University Hospital Zürich, Division of Hematology, Zürich, Switzerland; University of Massachusetts Medical School, United States of America

## Abstract

Stable integration of HIV proviral DNA into host cell chromosomes, a hallmark and essential feature of the retroviral life cycle, establishes the infection permanently. Current antiretroviral combination drug therapy cannot cure HIV infection. However, expressing an engineered HIV-1 long terminal repeat (LTR) site-specific recombinase (Tre), shown to excise integrated proviral DNA *in vitro*, may provide a novel and highly promising antiviral strategy. We report here the conditional expression of Tre-recombinase from an advanced lentiviral self-inactivation (SIN) vector in HIV-infected cells. We demonstrate faithful transgene expression, resulting in accurate provirus excision in the absence of cytopathic effects. Moreover, pronounced Tre-mediated antiviral effects are demonstrated *in vivo*, particularly in humanized Rag2^−/−^γc^−/−^ mice engrafted with either Tre-transduced primary CD4^+^ T cells, or Tre-transduced CD34^+^ hematopoietic stem and progenitor cells (HSC). Taken together, our data support the use of Tre-recombinase in novel therapy strategies aiming to provide a cure for HIV.

## Author Summary

Current antiretroviral combination therapy can efficiently suppress virus replication, but cannot eliminate HIV. Therefore, no cure for HIV exists. A main hurdle for virus eradication is seen in the existence of resting cells that contain integrated replication-competent, but temporarily silenced, HIV genomes. Therefore, the most direct approach to eliminating virus reservoirs is to remove HIV genomes from infected cells. As previous studies suggested, this may be achievable by Tre-recombinase, an engineered enzyme that can excise integrated HIV from host cell chromosomes. The present work analyzes the expression of Tre-recombinase in human cells and demonstrates highly accurate Tre activity in complete absence of Tre-related cytopathic effects. Furthermore, *in vivo* analysis of Tre-recombinase demonstrates highly significant antiviral effects of Tre in HIV-infected humanized mice. The presented data suggest that Tre-recombinase might become a valuable component of a future therapy that aims at virus eradication.

## Introduction

The introduction of highly active antiretroviral therapy (HAART) into clinical practice in the mid-1990s profoundly reduced morbidity and mortality among HIV-1-infected patients, changing an almost always fatal disease into a manageable chronic illness [1]. However, HAART is costly and occasionally not well tolerated [2], [3]. Particularly long-term HAART is frequently accompanied by emerging new toxicities, resulting in secondary complications that include metabolic disorders (e.g. diabetes, hyperlipidemia), osteoporosis, cardiovascular disease and chronic kidney disease (reviewed in [4]–[6]). Furthermore, large cohort studies demonstrated that the life expectancy of patients receiving HAART still remains considerably shorter than that of uninfected subjects (recently reviewed in [7]). Most importantly, the fact that HAART does not eradicate HIV and that treatment intensification, even when employing advanced drug regimens, fails to completely clear the virus (reviewed in [7], [8]) highlights the urgency of pursuing new strategies to find a cure for HIV infection.

It is generally believed that the main hurdle to virus eradication is the persisting HIV-1 infection in latent reservoirs, particularly in memory CD4^+^ T cells (reviewed in [9]–[15]). Latently HIV-1-infected resting CD4^+^ T cells are apparently established early in infection. One current strategy to eliminate this pool of long-lived cells aims to specifically activate the transcriptionally quiescent provirus (i.e. the integrated replication-competent HIV-1 genome), for example by modifying its chromatin structure through histone deacetylase (HDAC) inhibitors (reviewed in [12], [13], [15]–[17]). Upon HDAC inhibitor-induced HIV-1 antigen expression, it is expected that these cells either experience HIV-1-induced cell death or are eliminated by cytotoxic T cells (CTLs). It is fair to assume that such purging strategies would greatly benefit from a technology that can concurrently remove integrated HIV-1 from the pool of productively infected cells, thereby restoring, or at least improving the patient's immune function.

A novel strategy to remove integrated HIV-1 is based on a tailored site-specific recombinase (Tre), derived by molecular evolution of the bacteriophage recombinase Cre [18]–[20]. Tre targets a specific 34 bp sequence (loxLTR) derived from a primary HIV-1 strain [21] located in the proviral LTR regions, resulting in excision of the integrated proviral DNA from the genome of infected cultured cells [18]. This process not only suppresses viral replication, but in theory may also help eradicate HIV from an infected individual (reviewed in [22]).

Administering Tre-recombinase to patients will most likely require a gene therapy approach. In principle, genetic therapies against HIV either modify the patient's peripheral CD4^+^ T cells or patient-derived CD34^+^ hematopoietic stem cells (HSC) [23]–[25]. It is anticipated that the former strategy would lead to beneficial antiviral, although transient effects. The latter application will presumably be the preferred strategy in Tre-based virus eradication approaches, since, in theory, it allows perpetual repopulation of the patient's hematopoietic system with Tre-expressing HIV-1 target cells. These cells may be selected *in vivo* [26], since upon *de novo* infection they are able to remove the integrated HIV-1 proviral DNA, and thus remain functionally immune competent.

Independently of the selected gene therapy strategy, and prior to its potential use in HIV-infected patients, vector technology has to be developed that allows safe and efficient gene transfer followed by reliable transgene expression in target cells. Moreover, the absence of cytopathic and/or genotoxic effects upon vector-mediated Tre expression, and the accurate excision of HIV proviral DNA from chromosomal integration sites has to be demonstrated. Finally, the antiviral effects of Tre-recombinase have to be shown *in vivo*, i.e. in an appropriate animal model for HIV-1 infection. All these analyses will be of utmost importance for developing a potential Tre-based therapy to treat HIV infection.

## Results

### Conditional lentiviral vector-mediated Tre delivery

**Figure 1:**
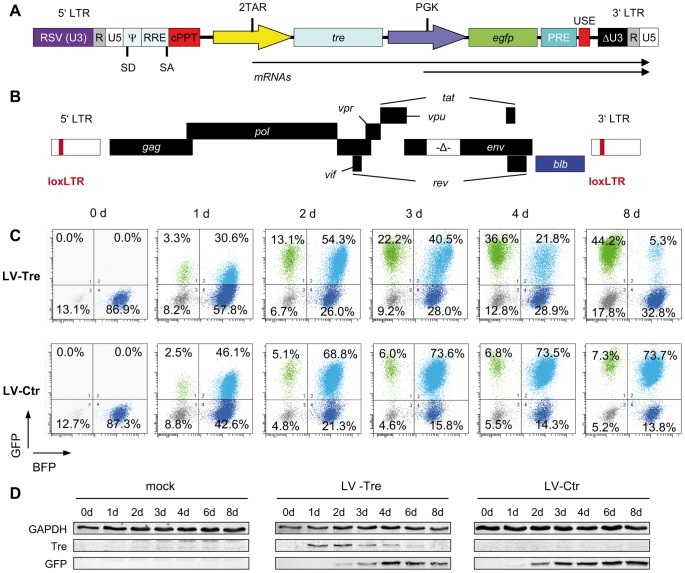
Tre-mediated antiviral activity by conditional transgene expression. (A) The HIV-derived lentiviral vector (LV) contains self-inactivating (SIN) long terminal repeats (LTR: ΔU3, R, U5), a Rev response element (RRE), central polypurine tract (cPPT), transgene cassette, post-regulatory element derived from woodchuck hepatitis virus (PRE), SV40 upstream polyadenylation enhancer elements (USE), splice donor (SD), splice acceptor (SA), and packaging (Ψ) signal, and the open reading frame for Tre-recombinase (*tre*) under the control of an HIV-LTR promoter containing two TAR elements (2TAR). In addition, the LV-Tre construct encodes the open reading frame for enhanced GFP (*egfp*). Expression of *egfp* is facilitated by the PGK promoter (PGK). (B) Schematic diagram of the replication–incompetent proviral reporter construct pNLT2ΔenvBLB. The open reading frame for the BFP-blasticidin-S deaminase fusion protein (*blb*) (blue box) partially substitutes the *nef* coding region. The 5′ and 3′ LTRs contain the Tre recombination site (loxLTR), a native HIV sequence. (C) Flow cytometric analysis of propidium iodide negative cells from one representative infection experiment follows the progression of GFP and BFP expression in polyclonal HeLa-smurf cells transduced with the indicated lentiviral constructs. (D) Tre and GFP expression was visualized by Western blot analysis. GAPDH served as a loading control.

For delivery of Tre-recombinase an HIV-1-derived replication-incompetent lentiviral vector (LV) was constructed that provides high safety levels due to a split packaging system; the self-inactivating (SIN) vector design; and a sequence element introduced to improve transcriptional termination [27], [28] (Figure 1A). To avoid transgene-related side effects, the gene sequence encoding Tre-recombinase was placed under the control of an engineered Tre-resistant tandem TAR repeat (2TAR), the *cis*-active target sequence of the HIV-1 Tat *trans*-activator [29]. This not only limits Tre expression to HIV-infected cells, but a duplicated TAR element also positively influences internal LTR promoter activity in the presence of Tat (Figure S1 in Text S1). Finally, constitutive expression of the GFP marker protein was facilitated by the PGK promoter (Figure 1A).

First, we investigated whether Tre-recombinase introduced by the LV construct is faithfully expressed in HIV-infected cells, and in turn, excises integrated chromosomal proviral DNA. We generated a reporter cell line, HeLa-smurf, which is stably infected with a replication-incompetent HIV-1 mutant with the *env* gene partially deleted and the *nef* open reading frame partially replaced by a marker gene (*blb*) (Figure 1B). Thus, Tre-mediated excision of the proviral genome results in loss of blue fluorescent protein (BFP) expression, which can be tracked by flow cytometry.

To monitor Tre activity, HeLa-smurf cells were transduced in triplicates with the GFP-expressing LV particles LV-Tre or a scrambled *tre* version, the Tre-negative control construct LV-Ctr, cultured for various time periods and analyzed for BFP and GFP expression. After transduction, both lentiviral constructs produced a GFP/BFP double positive cell population (see top right quadrants in Figure 1C, and plotted data in Figure S2 in Text S1). However, whereas the BFP/GFP double positive population of LV-Ctr-transduced cells subsequently remained stable over time, the BFP/GFP double positive population observed in LV-Tre treated cells started to decrease at 72 hours post transduction (Figure 1C and Figure S2 in Text S1). Simultaneously, the GFP-only positive population increased, suggesting that Tre-mediated excision of HIV-1 proviral DNA has occurred. Importantly, as the BFP/GFP double-positive population declined, expression of Tre-recombinase decreased, owing to the Tat-driven promoter providing temporal Tre expression (Figure 1D).

**Figure 2:**
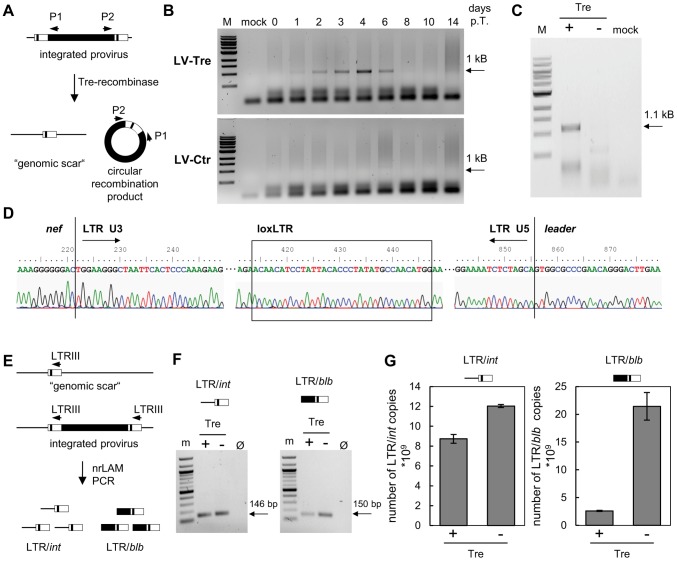
Tre-mediated provirus excision and determination of Tre efficacy. (A) Schematic depiction of Tre-mediated recombination. The PCR primers P1 and P2 amplify sequences from the provirus into the LTRs. Site-specific Tre-mediated recombination leaves a single LTR in the genome (“genomic scar”) and excises a circular recombination product containing the P1 and P2 primer binding regions. (B) Genomic DNA isolated from HeLa-smurf cells transduced with LV-Tre or LV-Ctr was analyzed by PCR using the P1 and P2 primers to detect the circular recombination product (1 kb). Negative PCR control, mock; lane M: DNA marker; p.t., post transduction. (C) To detect the genomic scar, genomic DNA prepared from Tre-treated (Tre +) or Ctr-treated cells (Tre −) 3 days after transduction were used as templates for PCR using HiLo PCR. Negative PCR control, mock; lane M: DNA marker. (D) The LTR region in the circular recombination product was subjected to DNA sequencing, revealing the presence of a single LTR flanked by *nef*-derived and *gag* leader sequences. Tre-treatment resulted in precise loxLTR recombination (boxed). (E) Genomic DNA containing either a single full length proviral genome or the residual LTR (“genomic scar”) is subjected to nrLAM PCR using LTR-specific primers (LTRIII). Subsequently, LAM PCR products consisting of LTR and genomic host DNA (LTR/*int*; 146 bp) or LTR and proviral coding sequence (LTR/*blb*; 150 bp) were quantified by qPCR. (F) Semi-quantitative PCR analysis of LAM PCR products from Tre-treated cells (Tre +), Ctr-treated control cells (Tre −) or negative PCR control (Ø). Lane M: DNA marker. (G) Quantitative PCR determination of LTR/*int* and LTR/*blb* LAM PCR products from Tre-treated cells (Tre +) or Ctr-treated (Tre −) control cells. Given are the means of three independent PCR reactions.

We performed a panel of assays to verify Tre-mediated excision of the provirus. First, we investigated the excised circular recombination product using a PCR assay (Figure 2A). An excision-specific fragment was only detected in DNA prepared from Tre-treated HeLa-smurf cells (LV-Tre), but not in cells treated with the negative control vector (LV-Ctr) (Figure 2B). The recombination product appeared as early as 24 hours after transduction, and can be detected until 6 days post transduction (p.t.) time point. As expected, the intensity of the PCR signal continuously increased, reaching a maximum at day 4 p.t., before starting to decline (Figure 2B). The direct sequencing of the LTR and its flanking regions confirmed that Tre-mediated recombination of loxLTR sequences had occurred in a highly accurate fashion, exactly maintaining the 34 bp loxLTR Tre-specific target sequence (Figure 2D). Importantly, the time-based occurrence and intensity of the excised proviral DNA closely corresponded with the declining BFP signal (Figure 1C and Figure S2 in Text S1), and coincided with temporal Tre expression (Figure 1D). Second, we only detected the remaining single LTR (i.e. genomic scar) in Tre-treated cells by PCR [30] (Figure 2C). Third, we identified a total of three proviral integration sites in genomic DNA from transduced HeLa-smurf cells by nrLAM PCR and high throughput sequencing [31] (Table S1 and Figure S3–Figure S5 in Text S1). As expected, proviral coding sequences (LTR/*blb*), as opposed to genomic sequences (LTR/*int*), were under-represented in Tre-treated cells compared to HeLa-smurf cells treated with the control vector as shown for one specific integration site by quantitative PCR (Figure 2E–G) as well as by integration site-independent next generation sequencing (Table 1). Thus, LV-Tre represents an efficient HIV-1 specific expression system for excising integrated HIV-1 provirus from cells.

**Table 1:** High throughput sequencing of nrLAM-PCR products.

	LV - Ctr	LV - Tre
**total # of mapped reads** A	4445	1009
**proviral coding region** B	1140 (25.6%)	165 (16.4%)
**integration site** C	3305 (74.4%)	844 (83.6%)

A number of quality-filtered reads that could be mapped to genomic or viral sequences (see Materials and Methods for details).

B,C number and percentages of reads mapping ^B^ to flanking/integration site specific sequences, or ^C^ to Nef-encoding proviral sequences.

### Absence of Tre-related cytotoxicity

**Figure 3:**
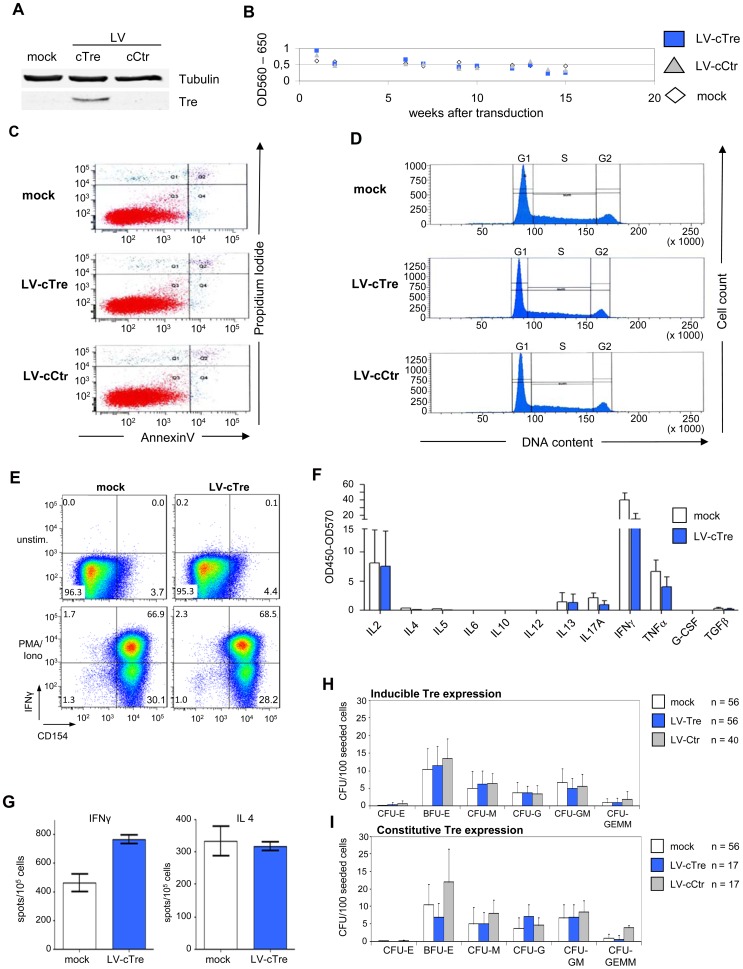
Analysis of potential Tre-related cytopathic effects. Exponentially growing Jurkat T cells were transduced with LV-cCtr, or LV-cTre encoding constitutively expressed Tre-recombinase, or mock transduced. After enriching GFP^+^ cells by FACS (LV-cCtr or LV-cTre), cells were cultured for up to 15 weeks. Every week cells were harvested and analyzed. (A) Western blot analysis of the indicated proteins using rabbit polyclonal anti-Tre serum and mouse anti-α-Tubulin antibodies. Protein signals were visualized using an Odyssey Infrared Imaging System (LI-COR). (B) Metabolic activity measured by MTT assay; (C) Apoptosis assessed by Annexin V assay at week 15 of constitutive Tre expression; (D) Cell cycle progression monitored by DNA staining at week 15 of constitutive Tre expression. (E) Functionality of human primary transduced CD4^+^ T cells tested by flow cytometric analysis of CD154 and IFNγ expression after PMA/ionomycin stimulation. (F) Secretion pattern of Th1-, Th2- and Th17-specific cytokines in primary transduced CD4^+^ T cells after PMA/ionomycin stimulation as determined by multiplex ELISA. The pattern matches that of non-transduced cells (mock). (G) IFNγ- and IL4-specific Elispot analysis of human primary transduced CD4^+^ T cells after PMA/ionomycin stimulation. (H) CD34^+^ HSC were transduced with LV-Tre (Tat inducible promoter configuration) and 100 cells were seeded into cytokine-containing methyl-cellulose. Culture dishes were incubated for 14 days at adequate conditions, before counting colonies. (I) HSC differentiation assay as in panel F using an LV constitutively expressing Tre-recombinase (LV-cTre).

The expression of antiviral genes may induce undesired effects, which could compromise host cell function. To analyze potential Tre-related cytotoxicities, Tre was overexpressed for a period of 15 weeks in Jurkat T cells; i.e. using the constructs LV-cTre and LV-cCtr in which the 2TAR promoter was replaced by the cellular EF1α promoter, permitting constitutive transgene (Tre) expression. Analyses of cellular metabolic activity (measured by an MTT assay), cell cycle progression (determined by DNA staining with propidium iodide) and apoptosis (assayed by Annexin V staining) did not reveal any deleterious effect of Tre expression on the host cells (Figure 3A–3D, respectively). This was also reflected by comparable cellular growth curves, independently of whether Tre-recombinase was expressed (Figure S6 in Text S1).

We also investigated possible effects of Tre expression on hematopoiesis and immune function. We transduced primary human CD4^+^ T lymphocytes with LV constitutively overexpressing the GFP marker protein and either Tre or the negative control. Subsequently, the GFP-positive cells (∼90% of the cultures) were analyzed with respect to immune activation by FACS, multiplex cytokine-ELISA and IL4 and IFNγ Elispot assays (Figure 3E–3G, respectively). These analyses suggest that prolonged overexpression of Tre does not negatively affect cellular activation of primary lymphocytes. We furthermore investigated the capacity of Tre vector-transduced CD34^+^ HSC to differentiate into various hematopoietic lineages using colony forming unit (CFU) assays. In all experiments, Tre vector-transduced HSC maintained their capacity to differentiate into the expected lineages (Figure 3H and 3I), with no significant differences from the controls.

**Figure 4:**
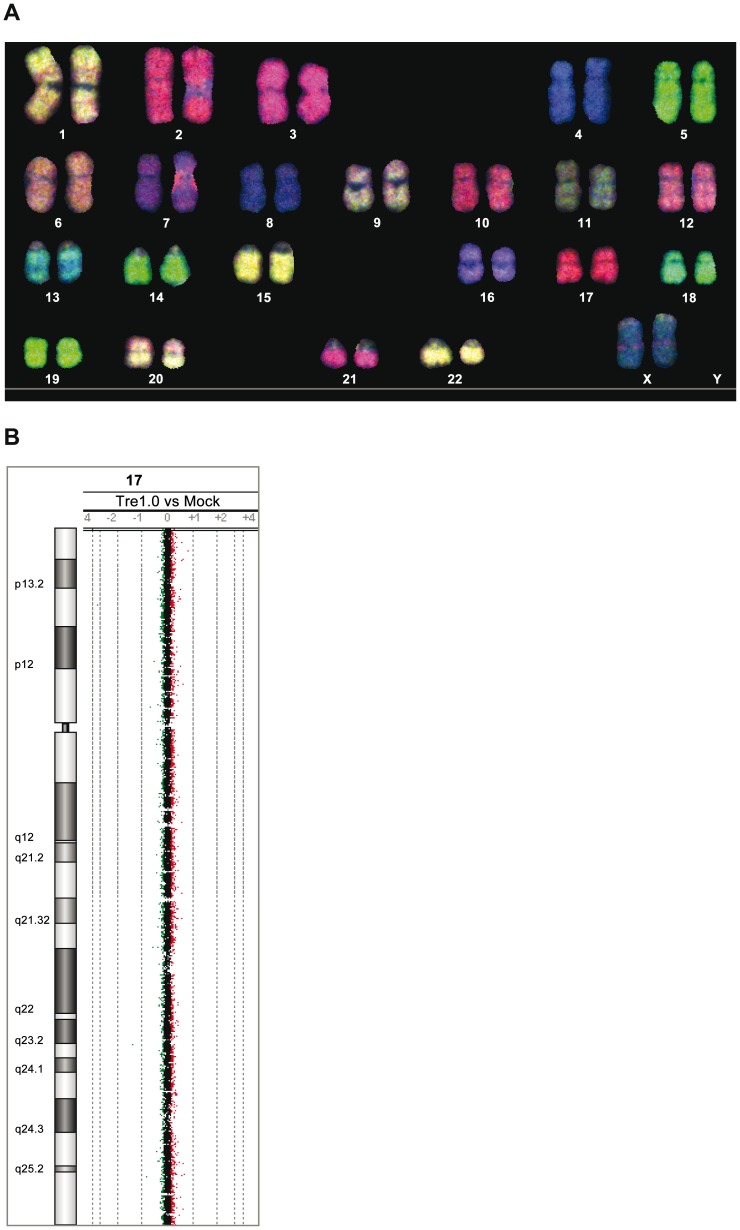
Analysis of potential Tre-related genotoxic effects. (A) SKY (spectral karyotyping) analysis of metaphase spreads isolated from primary human CD4^+^ T cells overexpressing Tre for 21 days. An RGB display of the 24-color SKY hybridization of a representative normal metaphase is shown. (B) Array-CGH analysis of DNA isolated from primary human CD4^+^ T cells overexpressing Tre compared to mock-transfected cells. A representative chromosome (Chr17) is shown. Normal log2 ratios of color intensities (−4 to +4) for each probe populate the chart. A heterozygous deletion would be indicated by a green dot with the value <−1. A heterozygous duplication would be indicated by a red dot with a value >0.66.

For further analyses, Tre was constitutively overexpressed for 21 days in primary human CD4^+^ T cells and potential chromosomal alterations were subsequently analyzed by spectral karyotyping (SKY) [32] and array-comparative genomic hybridization (array-CGH) [33]. The combination of these cytogenetic assays exhibited neither chromosomal translocations, nor variations in DNA copy-number changes (Figure 4A and 4B and Figure S7 in Text S1). Thus, Tre appears to lack any obvious off-target activity in human cells, at least within the limitations of these experimental systems.

**Figure 5:**
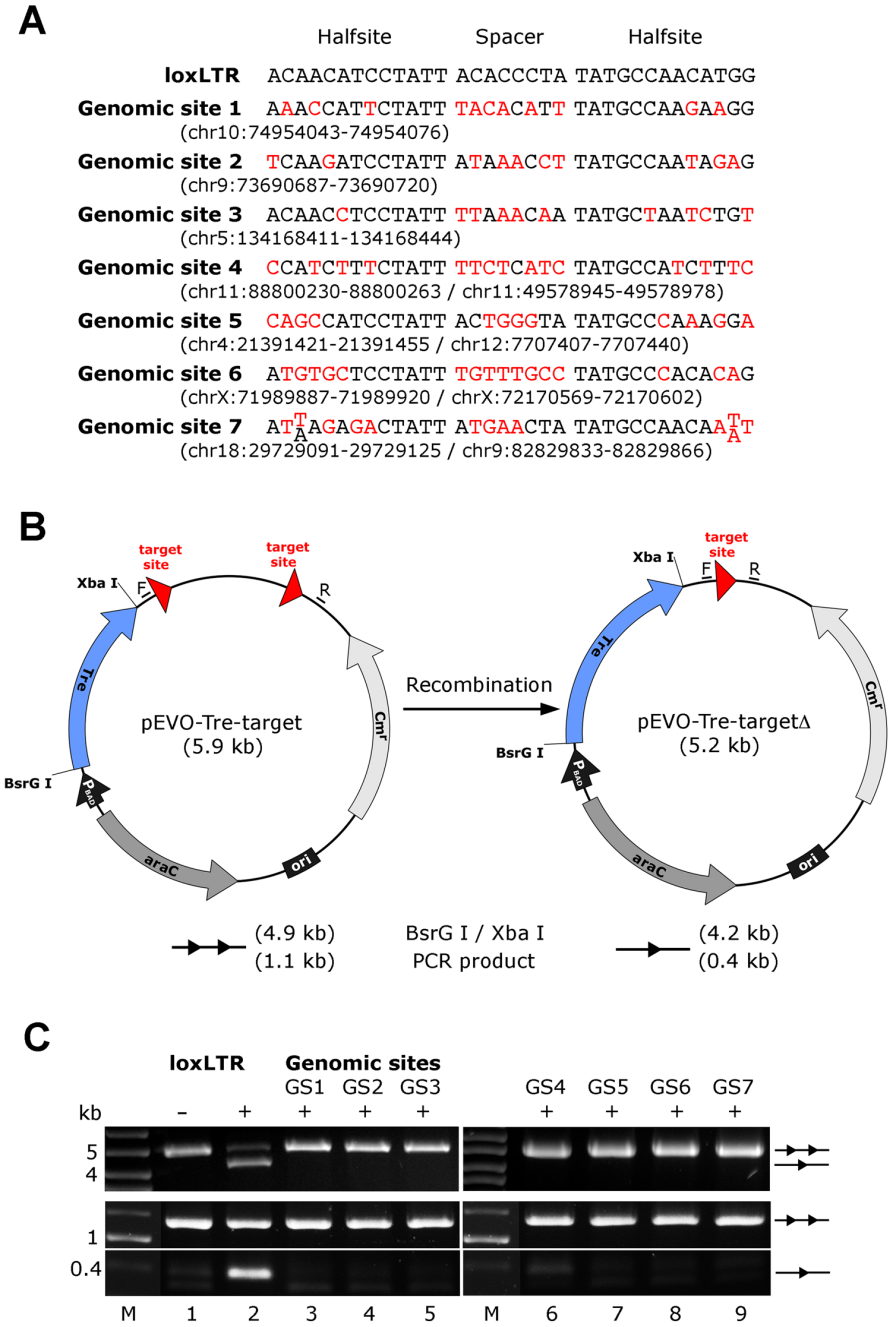
Assay of potential Tre-related off-target effects. (A) Nucleotide sequences of genomic sites and their locations in the human genome (in brackets). Sequences are aligned to the Tre recognition site loxLTR. Nucleotides that differ from loxLTR are shown in red. (B) Representation of the recombination assay in *E. coli*. and in HeLa cells, respectively. The evolution vector pEVO-Tre-target contains two directly repeated recombinase target sites (loxLTR) or the sequences GS1, GS2, GS3, GS4, GS5, GS6, and GS7. *In E. coli*, Tre is expressed from the P_BAD_ promoter upon induction with L-arabinose. The vector also contains the regulatory gene araC, and a chloramphenicol resistance marker (Cm^r^). Recombination at the target sites leads to deletion of the 700 bp intervening region. Locations of the PCR primer binding sites (F, R) for detection of recombination are indicated. (C) Agarose gel showing the activity of Tre on loxLTR and the lack thereof for the seven genomic sites GS1 to GS7 (lanes 3–9). Upper panel: Recombination assayed in *E. Coli*. BsrG I/Xba I restriction digest results in a 4.9 kb fragment for non-recombined plasmid (two triangles) and a 4.2 kb fragment for recombined product (one triangle). Recombination tests on loxLTR served as negative and positive control (lanes 1 and 2). −, non-induced; +, induced with 1 mg/ml L-arabinose; M, DNA marker lane. Lower panel: Recombination assayed in HeLa cells. PCR using primers F and R that anneal to the vector DNA results in a 0.4 kb product when recombination occurs, while the non-recombined template results in a 1.1 kb PCR product. −, cotransfection with pIRESneo (i.e. no Tre expression); +, cotransfection with pIRESneo-Tre [18].

Next we investigated other potential undesired alterations due to Tre expression. A recent transcriptome analysis using whole human genome microarrays found no differences between Tre-treated and untreated control cells [34], indicating that the expression of Tre does not alter the expression profile of cells. To verify Tre's target specificity, the SeLOX algorithm [35], a locus of recombination site search tool, was employed to scan the human genome for potential Tre target sites. Seven independent sequences that occur in the human genome and display the highest sequence similarity to loxLTR (5–8 nucleotide mismatches in the specificity-determining loxLTR halfsites) were tested as sites for Tre-mediated recombination in *E. coli* and HeLa cells (Figure 5). No recombination (above background signal) was detectable after strong and prolonged expression of Tre in *E. coli* or HeLa, respectively, demonstrating that naturally occurring human loxLTR-like sites are not a substrate for the recombinase. However, by screening the Los Alamos HIV sequence database (http://www.hiv.lanl.gov/), two additional independent clinical HIV-1 isolates were identified with subtle single nucleotide loxLTR variations that served as Tre targets (Method S1 and Figure S8 in Text S1). These findings suggest that Tre does not alter or recombine human chromosomal sequences (Figure 4 and Figure 5) but may recognize certain s\ingle nucleotide alterations in the loxLTR site of a variety of clinical HIV-1 isolates.

In conclusion, the combined data indicate that LV-mediated expression of Tre-recombinase does not induce cytopathic effects in human hematolymphoid cells.

### Tre-mediated antiviral activity in humanized mice

Two approaches, suggested as gene therapies against HIV [23], [24], were taken to test the ability of Tre to suppress HIV-1 infection *in vivo*. In the first approach, human CD4^+^ T cells were isolated from buffy-coats and transduced with LV-Tre or LV-Ctr particles, routinely resulting in ∼60% GFP^+^ cells (as measured by FACS; not shown). Then 6-week-old Rag2^−/−^γc^−/−^ mice were conditioned with clodronate, irradiated and transplanted with 3×10^6^ cells of the transduced total cell pools, which were characterized by CCR5 surface expression (Figure S9 in Text S1). Rag2^−/−^γc^−/−^ animals lack B, T, and NK cells, can be engrafted with either CD4^+^ T cells or CD34^+^ HSC, and in both cases, support HIV-1 infection [36]–[39].

The engraftment of human lymphocytes was verified at 8 to 10 weeks post transplantation by FACS analysis of PBMCs, determining the percentage of mouse CD45^+^, human CD45^+^, human CD4^+^, and GFP^+^ cells. To assess Tre activity, mice with ≥1% human CD45^+^CD4^+^GFP^+^ peripheral cells were then infected by intra-peritoneal injection of 100 ng p24 antigen of the replication-competent CCR5-tropic HIV-1 pNLT2env(BaL)mcherry, bearing loxLTR sites derived from the primary HIV-1 isolate TZB0003 [21]. Since we wanted to monitor Tre-mediated antiviral effects as directly as possible, i.e. by analyzing plasma viremia, this challenge virus does intentionally not encode a functional Nef protein. It is known that intact Nef depletes CD4^+^ T cells in humanized mouse models, thereby also indirectly affecting viral loads [40], [41]. Therefore, Nef-mediated pathogenic effects were not addressed in the following *in vivo* analyses.

**Figure 6:**
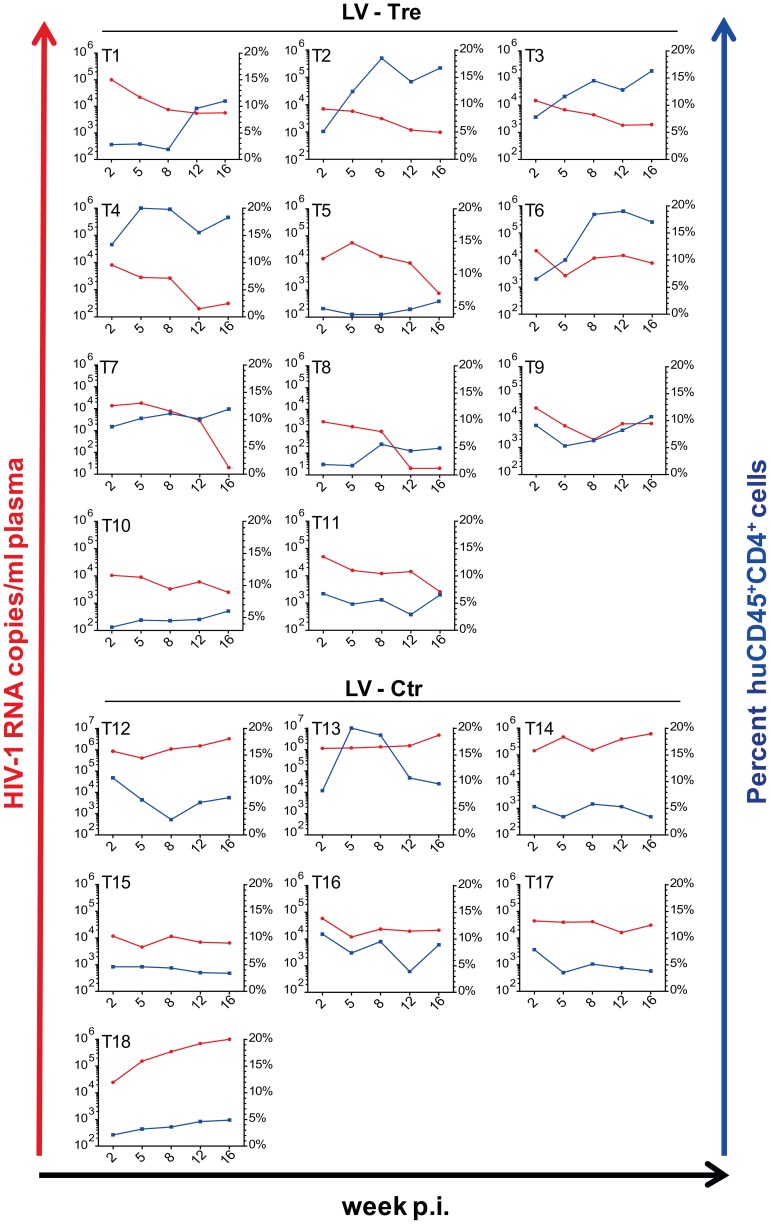
Suppression of plasma viremia in mice engrafted with Tre-modified human CD4^+^ T cells. Rag2^−/−^γc^−/−^ mice were infected with HIV-1 after adoptive transfer of LV-Tre (T1–T11) or LV-Ctr (negative control)-transduced (T12–T18) unselected CD4^+^ T cell pools. Plasma viral load (red lines) of individual mice was determined at the indicated time points after infection (p.i., post infection). Percent of human CD45^+^CD4^+^ cells in the peripheral blood of the individual animal is indicated (blue lines).

**Figure 7:**
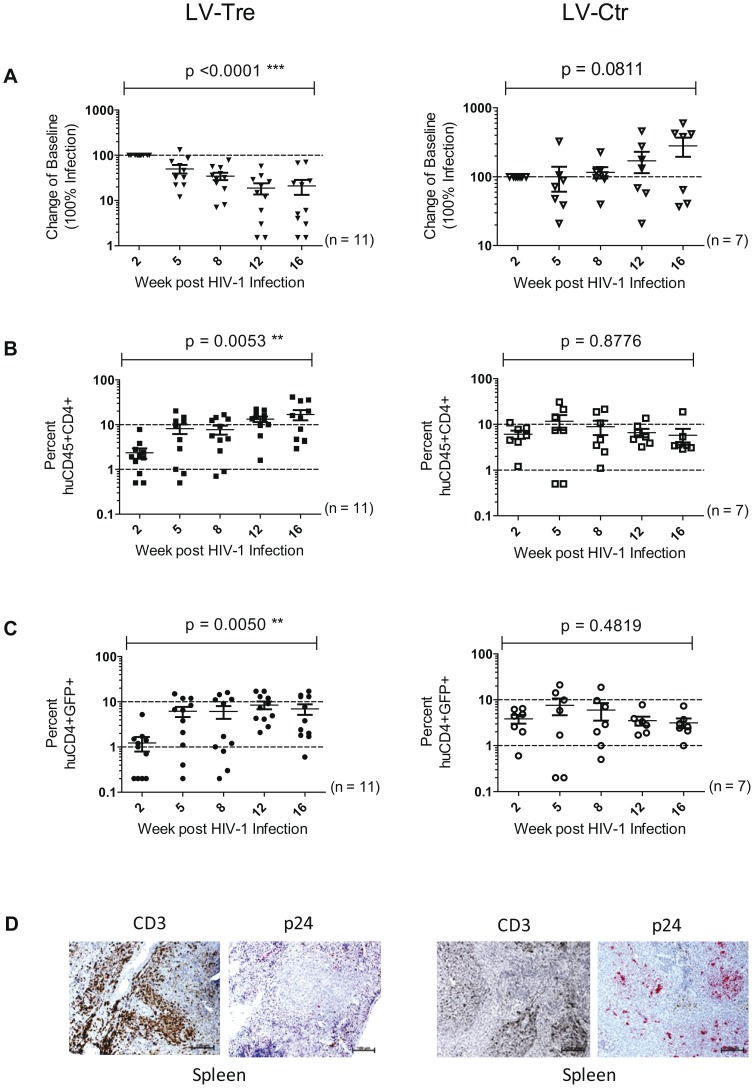
Analysis of the HIV-1 infected mice engrafted with LV-transduced (LV-Tre or LV-Ctr) unselected human CD4^+^ T cell pools. (A) Plasma viral load of individual mice at the indicated time points after infection is shown. The fold difference of change in baseline levels of HIV-1 RNA copies (set to 100%) in the mean (horizontal bars; paired two-tailed t-test), and the total number of animals analyzed in each cohort are indicated. (B) Flow cytometric detection of human CD45^+^CD4^+^ cells. (C) Lentiviral vector-derived GFP expressing human CD4^+^ cells in the peripheral blood of the animals (indicated in percent). (D) Immunohistochemical analysis of spleen sections prepared from mice engrafted with either LV-Tre (left panels) or LV-Ctr (right panels) transduced T cells and stained for human CD3 and HIV p24 antigen. Scale bars indicate 100 µm.

Upon infection, viral loads, GFP^+^ cells and PBMC surface markers (see Materials and Methods section) were subsequently monitored over time. Inspection of the data obtained from the individual animals revealed suppression of HIV-1 viremia in the plasma of the mice transplanted with Tre-transduced cells (LV-Tre; animal T1–11), but not in the negative control animals (LV-Ctr; animal T12–18; Figure 6). Moreover, in contrast to LV-Ctr treated animals, the percentage of human CD45^+^CD4^+^ cells increased during the 16 week observation period in the mice that received Tre-transduced CD4^+^ T cells (Figure 6). It should be noted that the engraftment of immune deficient mice with human cells is to a significant extent donor dependent, a fact that impacts on the animals' infection rates. Therefore, to obtain statistics, the HIV-1 RNA copies in the mice at week 2 post infection were set to 100% and the fold difference of the change in these baseline levels was followed over time. This analysis revealed a highly significant reduction of the viral load (p = <0.0001, n = 11; for statistical method see Materials and Methods section) in mice transplanted with Tre-transduced cells, as opposed to animals that received cells transduced with the negative control vector (p = 0.0811, n = 7) (Figure 7A). Moreover, the increase in the percentage of human CD45^+^CD4^+^ cells, particularly in Tre-treated mice as opposed to control animals was clearly significant (Figure 7B). This trend was also seen when assessing the percentage of CD4^+^GFP^+^ cells (Figure 7C). At 16 weeks after infection, animals were euthanized and various tissues analyzed. Immunohistochemistry on spleen sections clearly revealed reduced numbers of HIV-infected cells in a Tre-treated as compared to a control animal (Figure 7D). FACS analyses of single cell suspensions derived from bone marrow, liver, spleen and thymus showed an enrichment of CD4^+^GFP^+^ cells, particularly in animals that had received Tre-transduced cells (Figure S10 in Text S1).

**Figure 8:**
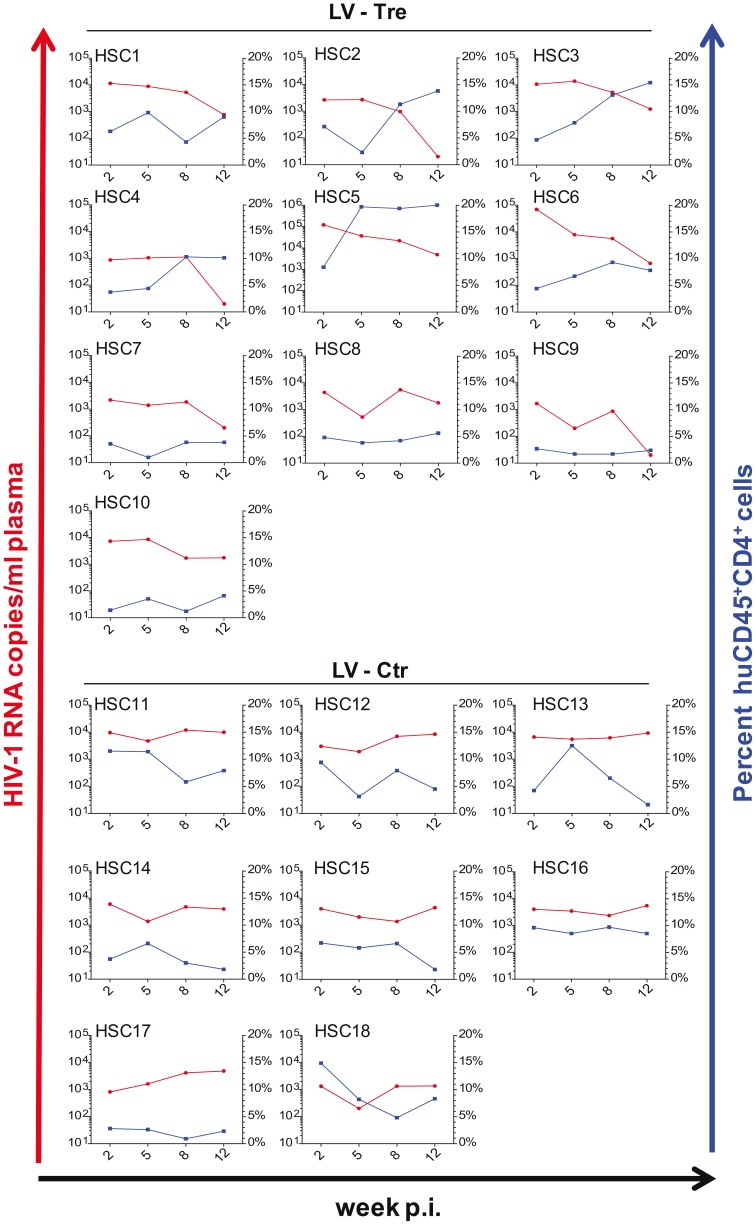
Tre-mediated antiviral effects in Rag2^−/−^γc^−/−^ mice engrafted with Tre-transduced human CD34^+^ HSC. Animals engrafted with LV-Tre (HSC1-HSC10) or LV-Ctr (negative control)-transduced (HSC11–HSC18) unselected cord blood-derived CD34^+^ HSC pools were infected with HIV-1 and analyzed over time. Plasma viral load (red lines) of individual mice was determined at the indicated time points after infection (p.i., post infection). Percent of human CD45^+^CD4^+^ cells in the peripheral blood of the individual animal is indicated (blue lines).

**Figure 9:**
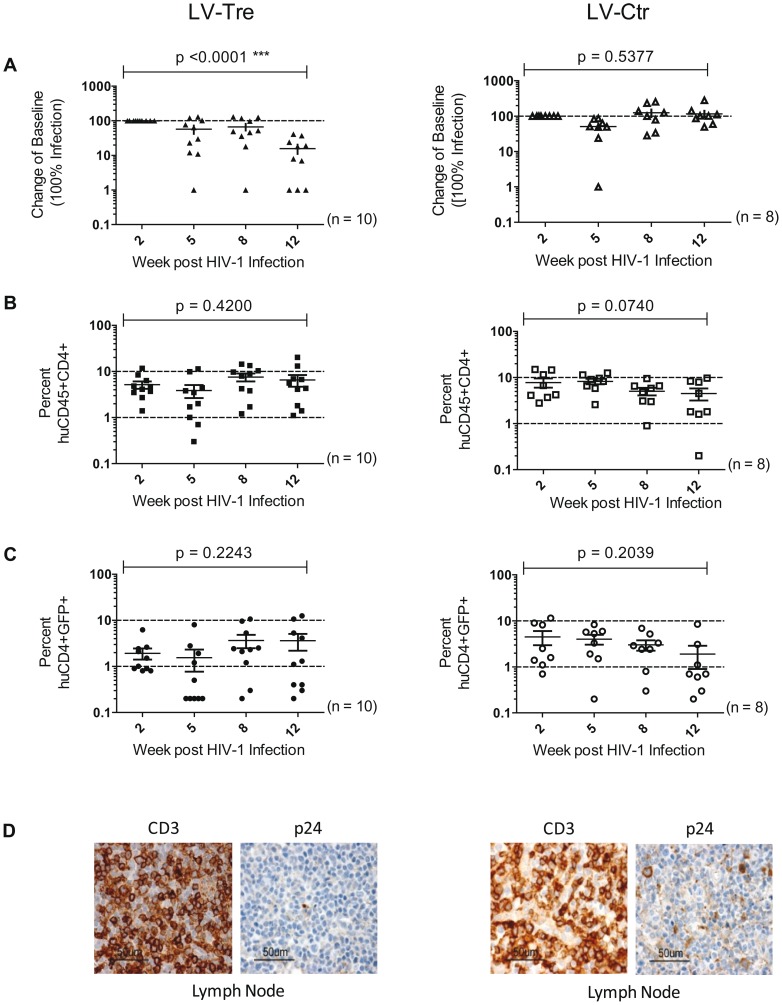
Suppression of plasma viremia in the Rag2^−/−^γc^−/−^ mice engrafted with Tre-modified (LV-Tre) CD34^+^ HSC. (A) Plasma viremia was determined at the indicated time points after infection of LV-Tre or LV-Ctr (negative control) transduced animals. The fold difference of change in baseline levels of HIV-1 RNA copies (set to 100%) in the mean (horizontal bars; paired two-tailed t-test), and the total number of animals analyzed in each cohort are indicated. (B) Human CD45^+^CD4^+^ cells and, (C) Transgenic GFP expressing human CD4^+^ cells in the peripheral blood of the animals (indicated in percent) were detected by flow cytometric analysis. (D) Immunohistochemical analysis of lymph node sections prepared from mice engrafted with either LV-Tre (left panels) or LV-Ctr (right panels) transduced HSC and stained for human CD3 and HIV p24 antigen. Scale bars indicate 50 µm.

In a second *in vivo* approach to study Tre-based antiviral effects, newborn Rag2^−/−^γc^−/−^ mice were irradiated and transplanted by intrahepatic injection with 3×10^5^ LV-Tre and LV-Ctr transduced human CD34^+^ HSC. The transduction rate of these cord blood-derived hematopoietic cells typically resulted in ∼30% GFP^+^ cells (not shown). Engraftment was verified at 10 to 12 weeks post transplantation by FACS analysis of PBMCs (determining the percentage of mouse CD45^+^, human CD45^+^, human CD19^+^, human CD3^+^, human CD4^+^ and GFP^+^ cells). Generally, animals with ≥0.5% of CD45^+^CD4^+^GFP^+^ lymphocytes in their peripheral blood were challenged with HIV-1 as described above. As shown, in all LV-Tre treated animals (HSC1–10), as opposed to mice that received LV-Ctr transduced HSC (HSC11–18), the individual viral load declined over time and the percentage of human CD45^+^CD4^+^ cells either increased or remained constant (Figure 8). Subsequent statistical analysis at week 12 post infection revealed that mean viremia in mice that received LV-Tre transduced HSC was significantly diminished (p = <0.0001, n = 10) compared to control (i.e. LV-Ctr) animals (p = 0.5377, n = 8) (Figure 9A), indicating a progressive loss of viral loads in Tre-treated animals over time. In contrast to the human CD4^+^ T cell transplanted animals, the percentage of CD45^+^CD4^+^ T cells did not change significantly in these mice (Figure 9B). This may be explained by the fact that only a fraction of the transplanted HSC (∼30%) were Tre-transduced, and thus protected from HIV replication. CD4^+^GFP^+^ cells were detected over the entire 12-week period, demonstrating the successful development of LV-transduced peripheral T cells in these HSC transplanted mice (Figure 9C). The immunohistochemical analysis of human CD3^+^ and HIV-1 p24 antigen expressing cells in lymph nodes of representative euthanized mice demonstrated that p24^+^ cells were distinctly depleted in mice that received LV-Tre transduced HSC (Figure 9D). Furthermore, FACS analysis of cell suspensions derived from bone marrow or spleen of these mice verified the presence of transgenic human cells representing various hematopoietic lineages, including CD4^+^ and CD8^+^ T lymphocytes, pre-B and activated B cells, cells committed to the monocyte/macrophage lineage, NK cells and NKT cells (see Figure S11 in Text S1).

Collectively, the *in vivo* experiments document the antiviral activity of Tre-recombinase at the organismal level.

## Discussion

The clinical development of HAART has been one of the great successes in modern medicine. However, the fact that HAART cannot eradicate HIV [7], [8] makes investigating novel antiviral strategies a prerequisite for developing a future cure for HIV infection [7], [9], [12]. In effect, gene therapy strategies represent a technology holding high promise for future antiviral disease treatments [22]–[25]. Indeed, various RNA-based technologies are currently being investigated *in vivo*, including, for example, the expression of RNA aptamers, siRNAs and shRNAs, TAR decoys, and ribozymes [42]–[44]. Moreover, the expression of membrane-bound fusion inhibitors is another appealing antiviral strategy [45], [46]. These approaches efficiently suppress virus replication, and thus reduce viral loads for extended periods of time. Another promising strategy appears to be disruption of the *CCR5* gene [47], [48], for example by expressing engineered zinc finger nucleases (ZFN) [20]. In humanized mice transplanted with either CD4^+^ T cells or CD34^+^ HSC, ZFN-mediated CCR5 disruption has been shown to confer resistance to *de novo* infection by CCR5-tropic HIV-1, thereby controlling virus replication [49], [50].

In contrast, an antiviral strategy based on Tre-recombinase is independent of virus coreceptor usage (i.e. tropism) and can target cells that are already infected with HIV [18], [34]. Importantly, Tre-mediated provirus excision allows reversal of HIV infection at the cellular level, thereby avoiding viral cytopathic effects (e.g. effects associated with viral antigen expression) and possibly restoring host cell function. As shown here, Tre expression mediated highly significant antiviral effects, which were equally observed in animals engrafted with Tre-expressing CD4^+^ T cells or Tre-expressing CD34^+^ HSC. With respect to future clinical studies, this Tre effect is particularly impressive, since the animals had been transplanted with unselected cell pools, where only a fraction of the T cells (∼60%) or the HSC (∼30%) harbored the Tre expressing lentiviral vector. Apparently, Tre-mediated protection of only a subpopulation of HIV-1 target cells suffices to achieve significant antiviral effects *in vivo*. This may be explained by the *in vivo* selection of gene vector-transduced cells as well as potential bystander effects [22]–[25].

The results presented here suggest that Tre vectors are promising antiviral reagents for therapies based on the genetic modification of both peripheral T cells and hematopoietic stem cells. Clearly, the procedure for *ex vivo* treatment of peripheral T cells is comparably less complicated, and aims at a functional cure by achieving long-term control of HIV, preferably in the absence of HAART [7]. In contrast, the development of a sterilizing cure that eradicates HIV-1 from an infected organism, if achievable at all, most likely requires a highly complex strategy, involving the autologous transplantation of gene-modified HSC [22], [25]. It is then expected that the elimination of all HIV-infected cells may eventually depend on reconstituting the patient's immune functions, a process that presumably requires additional and potentially gene therapy-unrelated approaches such as, for example, immune activation and/or purging strategies [51]–[54]. It is likely that such multi-pronged eradication approaches will benefit from Tre-mediated provirus excision in the patient's immune effector cells (e.g. CD4^+^ T cells).

Important safety issues related to gene therapies are generally connected with potential cytopathic effects caused by the respective transgene and/or the vector technology used. The latter was addressed here by using an advanced lentiviral SIN vector design where transgene expression was placed under the control of a Tat-inducible promoter, limiting its expression to HIV-infected cells. This strategy circumvents a major shortcoming of various antiviral gene therapies, the continued expression of foreign transgenes [23], thereby minimizing undesired transgene-related side effects such as immunogenicity.

Obviously, expression of Tre-recombinase from a Tat-inducible promoter presumably precludes provirus excision in latently infected cells. It is therefore conceived that in clinical virus eradication approaches Tat-responsive Tre-expressing vectors will only be used in combination with purging drugs that, as previously shown, not only specifically activate the transcription of otherwise quiescent proviral genomes [55], [56], but, at the same time, will also enable Tat-mediated Tre expression from the vector used in the present study. In this context it is also important to note that a recent study demonstrated that drug-induced purging alone does not result in the elimination of patient-derived infected resting CD4^+^ T cells, even when autologous CTLs were present [57]. In fact, after virus reactivation these cells where only killed when the HIV-specific CTLs were pre-stimulated, suggesting that virus eradication depends at least on a combination of purging drugs with therapeutic vaccination strategies [57]. It is expected that such an approach would further benefit from the inclusion of an additional anti-HIV gene therapy [25], [58], such as Tre-mediated provirus excision [22]. It is also noted that recent computational modeling of HIV dynamics in the presence of a replication incompetent Tre-recombinase-expressing therapeutic vector suggested that such an approach may indeed clear all HIV from the system in the long term [59].

Clearly, our study does not investigate the efficacy of Tre for latent proviruses. This is a significant limitation that will be addressed in the future. Particularly, it will be of interest to see whether a residual Tat level exists in latently infected cells that enables Tre expression by the current vector design. Alternatively, Tat-independent vectors that employ drug-inducible promoters may permit conditional Tre expression in resting cells. For example, advanced doxycycline-responsive promoter systems hold the promise to further increase biosafety of gene therapies by actively controlling transgene expression [60]. In this context one may also conceive the direct delivery of Tre-recombinase into patients, for example by applying Tre-containing virus like particles [61]. It is noted, that excision of proviral DNA by recombinant cell permeable Tre-recombinase has been already demonstrated in cell cultures [34]. Thus, such advanced Tre delivery systems are conceived to play an important role in the future, particularly for targeting latently infected resting cells.

Another safety aspect that should not be underestimated is based on Tre's pronounced target site specificity. The fact that the site-specific recombination process mediated by such Cre-derived enzymes neither produces free DNA ends (e.g. double-strand breaks) nor requires additional host factors [62], minimizes the oncogenic risk. In agreement, the advanced molecular cytogenetic analyses presented here demonstrate the absence of Tre-related genome-wide off-target effects. This distinguishes Tre-recombinase from CCR5-specific ZFNs, which may suffer from off-target cleavage specificities [49], [63], [64]. Nevertheless, virus entry inhibition by CCR5 knockout represents a highly attractive antiviral strategy that may be exploited to its full extent when combined with Tre-recombinase technology, thereby not only blocking *de novo* infection but also targeting already infected cells for provirus excision.

The presented data suggest that antiviral gene therapies are feasible using conditionally expressed, engineered Tre-recombinases that precisely remove HIV-1 proviral DNA without cytopathic effects. Antiviral *in vivo* activity was observed by transduction of both CD4^+^ T cells and CD34^+^ HSC. Particularly the latter stem cell-based approach may be a valuable component of future eradication strategies to cure HIV [22], [25]. The fact that the current Tre-recombinase recognizes particularly HIV-1 subtype A isolates may limit its broad application. However, the recent identification of highly conserved HIV-1 LTR sequences [65] in combination with a novel loxLTR search tool [35] now permits the engineering of advanced Tre-recombinases with activity against the majority of HIV-1 variants.

Clearly, it is not expected that HIV-1 can be eradicated by Tre activity alone. As outlined above, future HIV eradication strategies are conceived to be a combination of various antiviral approaches (e.g. drug-based and gene therapies), host immunity enhancing treatments (e.g. therapeutic vaccination approaches), and purging attempts to overcome latency [51]–[53]. In summary, our data support the notion that Tre-recombinase technology can be a valuable component of such a multi-tiered strategy to treat HIV-infected patients.

## Materials and Methods

### Ethics statement

The animal experiments were performed according to the guidelines of the German Animal Protection Law. The experimental protocols were reviewed and approved by the relevant German authority, the local ethics commission (Ärztekammer Hamburg; OB-050/07 and WF-010/2011) and the Freie und Hansestadt Hamburg, Behörde für Gesundheit und Verbraucherschutz (Nr.: 63/09 and 23/11).

### Generation of lentiviral vectors and proviral constructs

The lentiviral (HIV-1-based) SIN vector backbone has been described previously [28], [66]. Briefly, the vector comprises self-inactivating long terminal repeats (SIN LTRs) (ΔU3, R, U5), splice donor (SD), splice acceptor (SA), packaging signal (Ψ), central polypurine tract (cPPT), and the Rev response element (RRE). A post-regulatory element derived from woodchuck hepatitis virus (PRE) ensures efficient posttranscriptional RNA processing [67], [68] and a duplicated simian virus 40 (SV40) upstream polyadenylation enhancer element (USE) optimizes termination of transgene transcription [28].

The transgene expression cassette includes either the open reading frame of Tre-recombinase (*tre*) [18] or a scrambled version, plus sequences encoding the enhanced green fluorescent protein (*egfp*) [28]. The scrambled version serves as a negative control (Ctr); since all ATG start codons were replaced by TGA stop codons, and all GTG triplets by CCT, the respective mRNA cannot be translated into a protein. The *tre* and scrambled sequence are under the control of a Tre-resistant variant of the HIV-1 NL4-3 LTR (GenBank accession number M19921), containing one or two TAR elements. The TAR duplication (2TAR) was generated by standard PCR technology using HIV-1 NL4-3 proviral DNA as the template for amplification. Expression of *egfp* is under the control of the constitutive human phosphoglycerate kinase (PGK) promoter [27], resulting in a dual-promoter vector design.

The proviral construct pNLT2ΔenvBLB was generated by replacing the *Blp*I×*Xho*I fragment of construct pNLT2ΔenvPuro [18] with the coding sequence for a fusion protein (BLB) composed of mTag-BFP (Evrogen) and blasticidin-S deaminase [69], both linked by the linker sequence 5′-GCGCTAGGTGCTGCCGCCGGTGGT-3′.

The proviral plasmid pNLT2env(BaL)mCherry, encoding CCR5-tropic replication-competent HIV-1 was constructed by inserting the *env* gene (2572 bp) derived from plasmid pWT/BaL [70] (NIH AIDS Research & Reference Reagent Program, Cat.No. 11414) into the previously described vector pNLT2ΔenvPuro [18]. Puromycin resistance encoding sequences were replaced by the gene (711 bp) encoding the autofluorescent protein mCherry, which was derived from the plasmid pRSET-mCherry [71] provided by Dr. Roger Y. Tsien, University of California San Diego.

### Cell culture

HeLa and 293T cells were cultured at 37°C and 5% CO_2_ in Dulbecco's modified Eagle medium (DMEM; Biochrom) containing 100 units/ml of penicillin and streptomycin (PenStrep; Biochrom) and 10% fetal calf serum (FCS; Biochrom).

To generate HeLa-smurf cells, 2×10^5^ HeLa cells were infected with pseudotyped pNLT2ΔenvBLB (MOI 1). Cells were sorted for BFP-positive cells 1 and 3 weeks after infection and cultured in DMEM supplemented with 100 units/ml of PenStrep, 10% FCS and 5 µg/ml blasticidin (Invivogen).

### Production of viral particles

HIV-1 pseudotypes and lentiviral particles for infecting cultured cells were produced by transient cotransfection of 2×10^6^ 293T cells with the lentiviral or proviral plasmid and the respective packaging plasmids [27] using polyethylenimine (PEI) as a transfection reagent according to the manufacturer's protocol (Polysciences, Inc.). In detail, 6 µg of pNLT2ΔenvBLB and 1.5 µg of pCMV-VSV-G [72] were used for transfecting the proviral construct, or 6 µg of lentiviral vector, 1.5 µg of pRSV-Rev [27], 1.5 µg of pCMV-VSV-G [72] and 3 µg of pMDLg/pRRE [27]. At 72 hours post transfection viral supernatants were collected and passed through 0.2 µm pore size filters to ensure removal of any viral aggregates.

Titers of lentiviral particles and HIV-1 pseudotypes were determined as fluorescent forming units per ml (ffu/ml). This involved infecting 5×10^4^ 293T cells with different volumes of viral supernatant as described below. At 72 hours post transduction cells were harvested and analyzed by flow cytometry for GFP or BFP expression. Samples that contained 5 to 25% GFP or BFP positive cells were used to calculate viral titers.

Replication-competent HIV-1 for infecting humanized mice was produced essentially as before by transfecting 2×10^6^ 293T cells with 6 µg of the HIV-1 plasmid pNLT2env(BaL)mCherry. At day 3 post transfection, virus-containing supernatants were passed through 0.2 µm pore size filters, concentrated using a Centricon Plus-70 device (Millipore Corp), and adjusted with RPMI culture medium (without supplements) to 1 ng/µl of p24 antigen.

### Infection and transduction of cell cultures

Cells were infected with various amounts of virus in the presence of 1 µg/ml polybrene (Sigma-Aldrich) and spinoculated at 300× g for 10 min at ambient temperature. After spinoculation cells were cultivated at 37°C and 5% CO_2_. Medium was changed 8 h post infection.

For transduction of primary CD4^+^ T cells, cultures were pre-stimulated with CD3/CD28 magnetic beads (Invitrogen) for 24 h according to the manufacturer's instructions. After prestimulation, various amounts of virus were added in the presence of 2 µg/ml polybrene (Sigma-Aldrich) and the cells were spinoculated as described above. After 24 h of incubation at 37°C and 5% CO_2_, the transduction procedure was repeated. Prior to further analyses, transduced cells were cultured in the presence of 500 IU IL-2 for a further 3 days at 37°C and 5% CO_2_.

For transduction of CD34^+^ HSC, cultures were prestimulated with the cytokine cocktail StemSpan CC110 (Stem Cell Technologies) for 24 h. Virus was added to the cells, which were maintained in StemSpan SFEM (Stem Cell Technologies) supplemented with cytokine cocktail CC110 and the cultures were subjected to spinoculation as described before. After 24 h of incubation at 37°C and 5% CO_2_, the transduction procedure was repeated.

### Western blot analysis

Total protein was prepared and Western blot analysis was performed as described previously [73]. Rabbit polyclonal anti-Tre serum (Davids Biotechnologie), mouse anti-β-Tubulin (Sigma-Aldrich), polyclonal chicken anti-GFP (Novusbio), or polyclonal rabbit anti-GAPDH (FL-335; Santa Cruz) antibodies were used. Protein signals were quantified using an Odyssey Infrared Imaging System (LI-COR).

### Preparation and quantification of RNA

Total cellular RNA was prepared, reverse transcribed into cDNA and quantified by quantitative PCR as described previously [74].

To quantify glyceraldehyde-3-phosphate dehydrogenase (GAPDH) sequences the following primers were used: forward, 5′-GTCATCA ATGGAAATCCCATCA-3′; reverse, 5′-TGGTTCACACCCATGACGAA-3′; probe, 5′-(FAM)-TCTTCCAGGAGCGAGATCCCTC-(TAMRA)-3′.

### Tre functionality assay

24 hours before transduction, the HeLa-smurf cells' medium was changed to medium without blasticidin. Subsequently, 2×10^5^ HeLa-smurf cells were infected with VSV-G pseudotyped LV-Tre or LV-Ctr (MOI 7.5) as described above.

From infected cultures genomic DNA, protein, and RNA were prepared at different time points (24–336 h) after transduction and analyzed for genomic *gag*-levels, Tre expression, GFP expression, occurrence of the circular recombination product, and the genomic scar, as described above and below.

In addition, expression of BFP and GFP was monitored by FACS analysis using a FACSCanto II (Becton Dickinson) system equipped with 405, 488 and 635 nm lasers.

### PCR analysis of recombination in HeLa-smurf cells

Genomic DNA was isolated from eukaryotic cells using the QIAamp DNA Blood Mini Kit (QIAGEN GmbH). The circular recombination product generated by Tre-mediated recombination was detected as follows: 1 µg of genomic DNA was analyzed by PCR using 5′ Mastermix (5 Prime) with forward primer P2 (5′-GCTGCCCTCTGGTTATGTGTG-3′), binding in the *blb* sequence, and reverse primer P1 (5′-CTTAATACCGACGCTCTCGCAC-3′), binding in the *gag* sequence of pNLT2ΔenvBLB (PCR conditions: 1 cycle: 94°C for 2 min/56°C for 2 min/72°C for 2 min – 40 cycles: 94°C for 2 min/58°C for 1.5 min/72°C for 2 min - 1 cycle: 72°C for 10 min).

To detect the genomic scar, proviral integration sites were determined by HiLo-PCR [30]. In addition to the original protocol, a “nested” HiLo-PCR was performed with an aliquot of the first HiLo reaction to improve the yield of specific integration site fragments. The reaction conditions for the first and nested HiLo PCRs were as follows (50 µl total volume): 25 µl of Maxima Hot Start Green PCR Master Mix (2×) (Fermentas), and 1 pmol of HiLo primer or nested HiLo primer. HiLo PCR was carried out with 1 µg of genomic DNA from cell lines at the following conditions: 1 cycle: 95°C for 5 min – 25 cycles: 94°C for 1 min/65°C for 1 min/72°C for 3 min - 25 cycles: 94°C for 30 sec/37°C for 30 sec/72°C for 2 min – 1 cycle: 72°C for 5 min.

The nested HiLo PCR was carried out with 0.1 µl of the HiLo reaction under the following conditions: 1 cycle: 95°C for 5 min - 25 cycles: 94°C for 30 sec/65°C for 30 sec/72°C for 3 min - 15 cycles: 94°C for 20 sec/37°C for 30 sec/72°C for 2 min – 1 cycle: 72°C for 5 min. HiLo primers used in this study were: 5′-GAAATGCTAGGC GGCTGTCAAACCTCCACTCTA-3′ to amplify fragments upstream the HIV 5′-LTR, and 5′-TAGAGTGGAGGTTTGACAGCCGCCTAGCATTTC-3′ for fragments downstream of the HIV 3′-LTR. For the nested HiLo PCR the following primers were used: 5′-AGCACCATCCAAAGGTCAGT-3′ to amplify fragments upstream the HIV 5′-LTR, and 5′-AAGTAGTGTGTGCCCGTCTGTTG-3′ to amplify junction fragments downstream of the HIV 3′-LTR.

### Recombination efficiency assay using non-restrictive linear amplification-mediated PCR (nrLAM-PCR)

To enrich the number of genomic DNA/LTR junctions and proviral genome/LTR junctions, nrLAM-PCR was performed as described previously [31]. Briefly, 0.5 µg of genomic DNA was used as a template for linear amplification using 5′-biotinylated LTR-specific primer LTRI (5′-GATATCTGACCCCTGGCCCTG-3′). Biotinylated linear PCR products were immobilized on streptavidine-conjugated magnetic beads (Dynal-Invitrogen). Afterwards, a 5′-phosphorylated and 3′-modified (dideoxycytidine, ddC) linker-cassette ssDNAlinker (5′-CCTAACTGCTGTGCCACTGAATTCAGATCTCCCG GGTC-3′) was ligated to the 3′-end of the linear amplification product. Subsequently, the linear amplification product was amplified using two sets of nested primers. The first round of exponential amplification used 5′-biotinylated primer LTRII (5′-GTGTGTAGTTCTGCCAATC-3′) and primer LCI (5′-GACCCGGGAGATCTGAATTC-3′). Biotinylated double-stranded PCR products were immobilized on streptavidine-conjugated magnetic beads as before, and non-biotinylated complementary strands were eluted as substrate for further reaction. The second round of amplification was performed with primer LTRIII (5′-AGGGAAGTAGCCTTGTGTGTG-3′) and primer LCII (5′-GATCTGAATTCAGTGGCACAG-3′).

nrLAM-PCR products were then used as a template for quantitative PCR to determine the number of provirus/LTR and Chr11q13/LTR junctions. For quantification, SYBR green fluorescence was measured using the following sets of primers: 5′-CATGGAGCAATCACAAGTAGC-3′ and 5′-GTGGCTAAGATCTA CAGCTG-3′ (provirus/LTR junction); 5′-TTTAGTAGAGACAGGGTTTCACCATG-3′ and 5′-AGGGAAGTAGCCTTGTGTGTG-3′ (Chr11q13/LTR junction).

Semi-quantitative analysis was performed with the same set of primers under the following PCR conditions: 1 cycle: 98°C for 1 min – 19 cycles: 98°C for 10 sec/58°C for 30 sec/72°C for 45 sec - 1 cycle: 72°C for 5 min using Phusion Polymerase (Fermentas).

### High throughput sequencing

High throughput sequencing and data analysis were carried out as described previously [31]. Briefly, nrLAM-PCR products were amplified with bar-coded PCR primers fused to GS FLX-specific adaptors (for primer sequences see Table S1 in Text S1), pooled and subjected to pyrosequencing on a GS FLX sequencer (Roche), using adaptor primer A. Sequencing reads were sorted according to their multiple sequence identifier (MID) tags and quality filtered to eliminate all reads that did not match long terminal repeat (LTR) sequences at their 5′-end. We identified all reads that extended at least 20 nucleotides [31] beyond the LTR, and after trimming LTR sequences, matched the flanking sequences to both the human genome and pNLT2ΔenvBLB. We noted the number of reads that mapped to integration sites or the *blb*-encoding sequences of pNLT2ΔenvBLB to generate the data shown in Table 1. The BLAT alignment tool, described in [31], as well as the CLC Genomics Workbench package (CLCbio) were used to map sequence reads, and the UCSC genome browser (http://genome.ucsc.edu/) was used to visualize integration sites (Figure S3–Figure S5 in Text S1).

### Isolation of primary human cells

Isolation of CD4^+^ T cells from buffy coats was carried out using the RoboSep negative selection human CD4^+^ T cell enrichment kit in conjunction with a RoboSep automated cell separator (StemcellTechnologies) according to the manufacturer's instructions. Likewise, the preparation of CD34^+^ HSC from umbilical cord blood was performed with the EasySep human cord blood CD34^+^ selection kit (StemcellTechnologies) and the RoboSep system.

### Analysis of cellular toxicity

MTT assay was performed with 100 µl of cell suspension and the MTT kit (Roche) according to the manufacturer's instructions. A VersaMax micro plate reader (Molecular Devices) was used for colorimetric assay evaluation.

Analysis of apoptotic events was performed using the Annexin V FITC kit (Invitrogen) together with the antibody Annexin V-APC conjugate (Becton Dickinson). For analysis, 5×10^5^ cells were harvested, stained according to the manufacturer's protocol, and analyzed using a BD FACSCanto II (Becton Dickinson) system.

To determine cell cycle distribution 1×10^6^ cells were harvested, washed with PBS, suspended in 500 µl PBS/1% EDTA and fixed drop-wise with 5 ml of 80% ice cold ethanol. After incubating for 20 min on ice, the cells were incubated for 24 h at −20°C. Afterwards, the cells were pelleted and rehydrated in 450 µl of PBS supplemented with 16.6 µl RNAse A (10 mg/ml; Sigma-Aldrich) and 33 µl propidium iodide solution (0.5 mg/ml; Sigma-Aldrich). Incubation at 37°C for 30 min, was followed by further incubation at ambient temperature in the dark for 2 h prior to flow cytometry using a BD FACSCanto II system.

### Immune activation analysis of transduced CD4^+^ T cells

Primary human CD4^+^ T cells were stimulated for 12–24 h with phorbol myristate acetate (PMA) (50 ng/ml final conc.) and ionomycin (0.67 µM final conc.). Specific cytokine levels were monitored by ELISA, Elispot and intracellular cytokine staining (ICS).

Human Th1, Th2, Th17 Cytokine Multi-Analyte ELISArray (Qiagen) was performed with supernatants from 1×10^6^ stimulated cells or unstimulated controls, according to the manufacturer's instructions. ICS was essentially performed as previously described [75] with the modification that monensin (Biolegend) was used to inhibit secretion. Mouse anti-human CD3-APC H7 (BD Biosciences), mouse anti-human CD4-APC (Becton Dickinson) and mouse anti-human CD154-PE (BD Pharmingen) antibodies were used for surface staining according to the manufacturer's instructions, except that a 4-fold excess of the CD154 antibody was directly added to the cells during stimulation. For intracellular staining, mouse anti-human IFNγ-PE-Cy7 antibody (BD Pharmingen) was used. Live/dead staining was performed in parallel using the LIVE/DEAD Fixable Aqua Dead Cell Stain Kit for 405 nm excitation (Life Technologies).

Elispot analysis was essentially performed as previously described [76]. Briefly, polyvinylidene plates (96-well; Millipore) were coated with 50 ng of recombinant anti-human IFNγ antibody (Mabtech), or 50 ng of recombinant anti-human IL4 antibody (Mabtech) in phosphate-buffered saline at 4°C for 12 h. Afterwards, 3×10^3^ to 1×10^5^ cells were seeded on the coated plates and stimulated with PMA/ionomycin as indicated above. Secreted IL4 or IFNγ was detected using the biotinylated detection antibodies anti-human IL4 (Mabtech) or anti-human IFNγ (Mabtech).

### Colony forming unit (CFU) assays

The differentiation potential of transduced HSC cells was performed with methocult H4435-enriched methylcellulose (Stem Cell Technologies) according to manufacturer's protocol. For this, 100 transduced or mock treated cells were suspended in 1 ml of methylcellulose and seeded into a 3.5 cm diameter cell culture dish (Stemcell Technologies). After incubation at 37°C and 5% CO_2_ for 14 days, various cell colonies were identified and counted.

### Spectral karyotyping (SKY) assay

Tre overexpressing primary human CD4^+^ T cells were arrested in mitosis 21 days post transduction by treating the cells with 0.1 µg/ml colcemid for 4 hours. Cells were then treated with 75 mM KCl, incubated at 37°C for 15 min and fixed in 75% methanol/25% acetic acid. Cell suspension was dropped onto glass slides. Metaphase chromosomes were hybridized with the SKY probe mixture and analysed as previously described [77] using the SpectraCube system (Applied Spectral Imaging) coupled to an epifluorescence microscope (Leica).

### Array-comparative genomic hybridization (array-CGH) analysis

Lentiviral transduced Tre expressing primary human CD4^+^ T cells were harvested 21 days post transduction and genomic DNA was extracted using the QIAamp DNA Blood Mini kit (Qiagen) for array-CGH analysis. DNA was hybridized against DNA from mock-transfected cells on an Agilent SurePrint G3 Human CGH Microarray Kit 2×400K. The minimum number of probes affected to designate an aberration was set to 3. The median over all probe spacing was 5.3 kb (4.6 kb in RefSeq genes) on the array used.

### Off-target recombination analysis

Potential off-target Tre recombination sites were identified by screening the human genome using the bioinformatics tool, SeLOX [35]. The respective genomic sites were cloned into the recombination reporter plasmid pEVO-Tre-target [18]. In *E. coli*, recombinase expression was induced with L-arabinose (Sigma-Aldrich) at 1 mg/ml. Plasmid DNA was isolated from overnight cultures and digested with *BsrG*I and *Xba*I (NEB), resulting in different fragment sizes for recombined versus non-recombined substrate on agarose gels. Recombination on the Tre target loxLTR served as positive control. In eukaryotic cell culture, HeLa cells were cotransfected with the reporter plasmids and the expression plasmid pIRESneo-Tre [18]. DNA was isolated from the cells 48 h post transfection and analyzed for recombination by polymerase chain reaction using the primers F: 5′- GACAATAACCCTGATAAATGC-3′, and R: 5′-CCTTAAACGCCTGGTGCTAC-3′.

### Generation of humanized Rag2^−/−^γc^−/−^ (hu-Rag2) mice

Humanized Balb/c Rag2^−/−^γc^−/−^ (provided by M. Ito, Central Institute for Experimental Animals, Kawasaki, Japan) were bred and maintained under specific pathogen-free conditions using individually ventilated cages (IVC).

To generate human T cell transplanted Rag2^−/−^γc^−/−^ mice, 6 week old animals were preconditioned by intra-peritoneal (i.p.) injection of 100 µl of clodronate liposomes (obtained from Dr. N. van Rooijen, Department of Molecular Cell Biology, Amsterdam, Netherlands). Twenty four hours later, animals were irradiated using a dose of 2×2 Gy (6 h and 2 h before transplantation) from a Cesium 137 source at 3.75 Gy/min (CSL-12; Conservatome). Subsequently, mice were transplanted with 3×10^6^ lentiviral vector (LV-Tre or LV-Ctr) transduced human CD4^+^ T cells in 150 µl PBS containing 0.1% human AB serum (PAN Biotech GmbH) by i.p. injection. Analysis of human cell engraftment was verified by FACS analysis of peripheral blood samples at 8 to 10 weeks post transplantation, using retro-orbital sampling. Likewise, following HIV-1 infection blood samples were analyzed every second to fourth week for a period of 4 months.

Animals transplanted with human hematopoietic stem/progenitor cells (CD34^+^ HSC) were generated by injecting newborn Rag2^−/−^γc^−/−^ mice 24 h after birth intra-hepatically (i.h.) with 3×10^5^ lentiviral vector (LV-Tre or LV-Ctr) transduced CD34^+^ cells in 30 µl PBS containing 0.1% human AB serum. Prior to i.h. injection, the newborns were irradiated with 2×2 Gy as before. Engraftment was verified by FACS analysis of peripheral blood samples at 10 to 12 weeks post transplantation and, following HIV-1 infection, every second to third week for a period of 3 months.

### HIV-1 infection of humanized mice

CD4^+^ T cell or CD34^+^ HSC transplanted mice were infected by i.p. injection of 100 ng p24 antigen (10^8^ HIV-1 RNA copies) of CCR5-tropic HIV-1 pNLT2env(BaL)mcherry, containing loxLTR Tre-recombinase target sites. Animals were bled from the retro-orbital venous sinus two weeks post infection, followed by collection of blood every second, third or fourth week. Viremia was assayed by diluting cell-free mouse plasma with human serum (PAN Biotech GmbH) using the ultrasensitive (<20 HIV-1 RNA copies/ml) Cobas AmpliPrep/Cobas TaqMan HIV-1 Test version 2.0 (Hoffmann-La Roche Ltd.).

### Analysis of human cells in mouse tissues

For analysis of peripheral cells, 50 µl to 100 µl of blood was collected from the retro-orbital venous sinus (r.o.) into 100 µl bleeding-buffer (PBS plus 10 mM EDTA) and red blood cells were lysed by treatment with Red Blood Cell Lysing Buffer (Sigma-Aldrich). The white blood cell pellet was resuspended in FACS-buffer (PBS containing 2% FCS and 2 mM EDTA) and stained with monoclonal antibodies.

Single cell suspensions of various organs (thymus, spleen, liver and bone marrow) for antibody staining and FACS analysis were prepared at necropsy by manual tissue dissection and filtering through a sterile 70 µm nylon mesh (BD Biosciences).

Stained cells were analyzed in FACS-buffer plus 1% paraformaldehyd using a FACSCanto II (Becton Dickinson) system with BD FACSDiva Software v5.0.3 and FlowJo software v7/9 for PC (Treestar). To monitor human cell engraftment, r.o. collected cells were stained with monoclonal antibodies raised against mouse CD45.2 (104) and human CD45 (H130), human CD3 (UCHT1), and human CD19 (HIB19) (all from eBioscience Inc.). The transduction rate was monitored by vector-derived GFP expression. HIV-1 infected mice were analyzed by staining with monoclonal antibodies directed against the human antigens CD45 (H130), CD4 (RPA-T4) (both from eBioscience Inc.), CD3 (UCHT1), CD8 (B9.11) (both from Beckman Coulter Inc.), CCR5 (3A9) and CXCR4 (12G5) (both from BD Pharmingen). Isotype antibodies and cells obtained from non-transplanted mice served as negative staining controls.

### Immunohistochemical analysis

Formalin fixed and wax embedded sections were analyzed. Deparaffinized sections were incubated in citrate buffer in an 85°C waterbath overnight for human CD3 antigen detection. Monoclonal anti-human CD3 (Dako M7254, clone F7.2.38) was used in a 1∶1000 dilution. Biotinylated anti-mouse monoclonal antibody in combination with horseradish peroxidase streptavidin was used for visualisation. The TNB-Amplification Kit (Dako) and diaminobenzidine were used as substrates. Sections were counterstained with haemalumn.

To visualize p24 antigen, the monoclonal antibody clone Kal-1 (Dako) was used. Deparaffinized sections were boiled for 20 min in retrieval buffer S 1699 (Dako) using a pressure cooker set at 100°C. Streptavidin alkaline phosphatase and the TNB-Amplification Kit with Permanent Red were used for visualisation.

For staining mesenteric lymph nodes, detected in HSC transplanted Rag2^−/−^γc^−/−^ mice, sections were incubated in titrated concentrations of mouse monoclonal anti-HIV p24 (Kal-1; Dako) and anti-human CD3 (SP7; Thermo Scientific) antibodies using an automated Ventana Discovery Module (Ventana Medical Systems). Stainings were developed according to the manufacturer's protocol as described previously [38].

### Statistical analysis

Significant values between the initial analysis (at week 2 post HIV-1 infection) and the final analysis (at week 12 or 16 post HIV-1 infection) within the LV-Tre animal group and the LV-Ctr animal group were calculated using the Student's paired two-tailed t-test of the GraphPad Prism Program version 5.03 (GraphPad Software). The two-tailed *p* values less than 0.05 were considered significant.

## Supporting Information

Text S1 Text S1 describes the method analyzing the activity of Tre against LTR sites of different HIV-1 strains and provides supporting figures demonstrating the Tat responsiveness of LV constructs, data plots of Tre analysis in HeLa-smurf cells, mapping of HIV integrations sites in HeLa-smurf cells, cellular growth curves upon Tre expression, detailed array-CGH analysis of the human chromosomes, Tre activity testing on different HIV-1 isolates, analysis of HIV-1 coreceptor expression in LV-transduced CD4^+^ T cells, and FACS analyses of single cell suspensions derived from various organs of Tre-transduced and HIV-infected mice. Finally, the sequences of bar-coded fusion primers used for pyrosequencing are provided.
(PDF)
