# Supplementary material for: Highly Significant Antiviral Activity of HIV-1 LTR-Specific Tre-Recombinase in Humanized Mice
Source: PLoS Pathog. 2013 Sep 26;9(9):e1003587. doi: 10.1371/journal.ppat.1003587 (PMC3784474; doi:10.1371/journal.ppat.1003587)
Supplement: Text S1 — describes the method analyzing the activity of Tre against LTR sites of different HIV-1 strains and provides supporting figures demonstrating the Tat responsiveness of LV constructs, data plots of Tre analysis in HeLa-smurf cells, mapping of HIV integrations sites in HeLa-smurf cells, cellular growth curves upon Tre expression, detailed array-CGH analysis of the human chromosomes, Tre activity testing on different HIV-1 isolates, analysis of HIV-1 coreceptor expression in LV-transduced CD4+ T cells, and FACS analyses of single cell suspensions derived from various organs of Tre-transduced and HIV-infected mice. Finally, the sequences of bar-coded fusion primers used for pyrosequencing are provided. (PDF) [file ppat.1003587.s001.pdf]

Text S1 - Supporting Information

Highly Significant Antiviral Activity of HIV-1 LTR-Specific Tre-Recombinase in Humanized Mice

Ilona Hauber[1], Helga Hofmann-Sieber[1], Jan Chemnitz[1], Danilo Dubrau[1], Janet Chusainow[2], Rolf Stucka[3], Philip Hartjen[1,4], Axel Schambach[5,6], Patrick Ziegler[7,8], Karl Hackmann[9], Evelin Schröck[9], Udo Schumacher[10], Christoph Lindner[11], Adam Grundhoff[1], Christopher Baum[5], Markus G. Manz[7,12], Frank Buchholz[2], Joachim Hauber[1]

[1]Heinrich Pette Institute – Leibniz Institute for Experimental Virology, Hamburg, Germany. [2]Department of Medical Systems Biology, University Hospital and Medical Faculty Carl Gustav Carus, TU Dresden, Dresden, Germany. [3]Friedrich-Baur-Institute, Department of Neurology, Ludwig-Maximilians-University Munich, Munich, Germany. [4]Infectious Diseases Unit, I. Department of Internal Medicine, University Medical Center Hamburg-Eppendorf, Hamburg, Germany. [5]Institute of Experimental Hematology, Hannover Medical School, Hannover, Germany. [6]Division of Hematology/Oncology, Children's Hospital Boston, Harvard Medical School, Boston, Massachusetts, USA. [7]Institute for Research in Biomedicine, Bellinzona, Switzerland. [8]Klinik für Onkologie, Hämatologie und Stammzelltransplantation, RWTH Aachen University, Aachen, Germany. [9]Institute for Clinical Genetics, University Hospital and Medical Faculty Carl Gustav Carus, TU Dresden, Dresden, Germany. [10]Institute for Anatomy and Experimental Morphology, University Cancer Center Hamburg, University Medical Center Hamburg-Eppendorf, Hamburg, Germany. [11]Department of Gynecology, DKH Hospital, Hamburg, Germany. [12]University and University Hospital Zürich, Division of Hematology, Zürich, Switzerland.

Method S1.

Assay of Tre activity against LTR sites of different HIV-1 strains

HIV-1 strains as potential targets for Tre were identified using the Los Alamos HIV sequence database (http://www.hiv.lanl.gov/). The respective loxLTR like target sites were cloned into the recombination reporter plasmid pEVO-Tre-target [18]. Recombinase expression was induced in *E. coli* with L-arabinose (Sigma) at 1 mg/ml. Plasmid DNA was isolated from overnight cultures and digested with *BsrGI* and *XbaI* (NEB), resulting in different fragment sizes for recombined versus non-recombined substrate on an agarose gel. Recombination on the Tre target site loxLTR served as positive control.

Supporting Figures

Figure S1. Tat responsiveness of LV constructs. HeLa cells stably transduced with the indicated lentiviral vectors (LV1TAR, LV2TAR) were cultured for seven days before being transiently transfected with an expression plasmid constitutively expressing the HIV-1 Tat protein under the control of an immediate early CMV promoter (Tat). Tre expression was monitored and quantified by Western blot analysis. β-Tubulin levels were used as normalization controls.

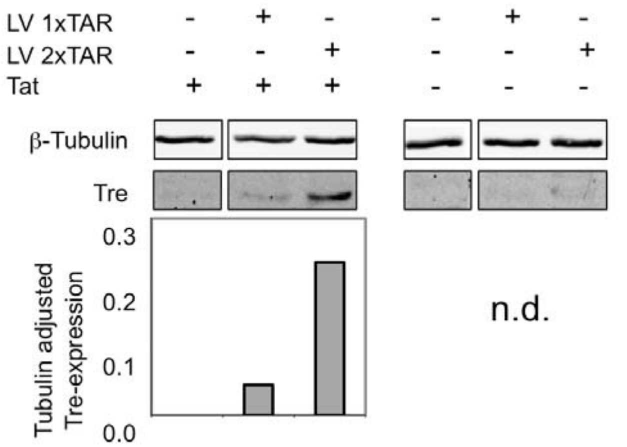

Figure S2. Data plots of Tre analysis in HeLa-smurf cells. GFP and BFP expression were plotted in cell populations transduced with the indicated lentiviral vectors. Mean values of propidium iodide negative cells of three independent infection experiments are shown; dark blue bars: GFP^-/BFP^+ cells; green bars: GFP^+/BFP^- cells; light blue bars: GFP^+/BFP^+ cells; black bars: GFP^-/BFP^- cells.

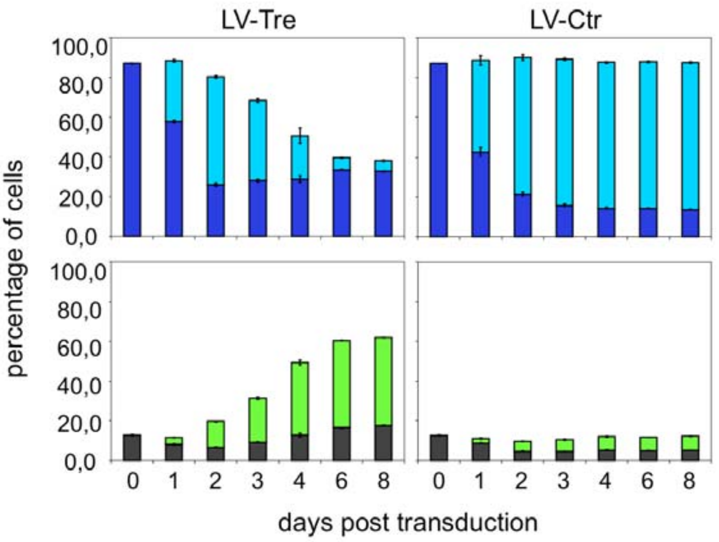

Figure S3. Integration site on chr16q12.2. The integration site on chromosome 16, q12.2 mapped by high throughput sequencing of nrLAM-PCR products. The site is located in the sixth intron of the RPGRIP1L (RPGRIP1-like, Gene ID: 23322) gene, co-linear with the direction of transcription (upper panel). The lower panel shows the genomic sequences flanking the integration site, with nucleotides covered by the longest read (chr16: 53,709,758-53,709,838) highlighted in blue.

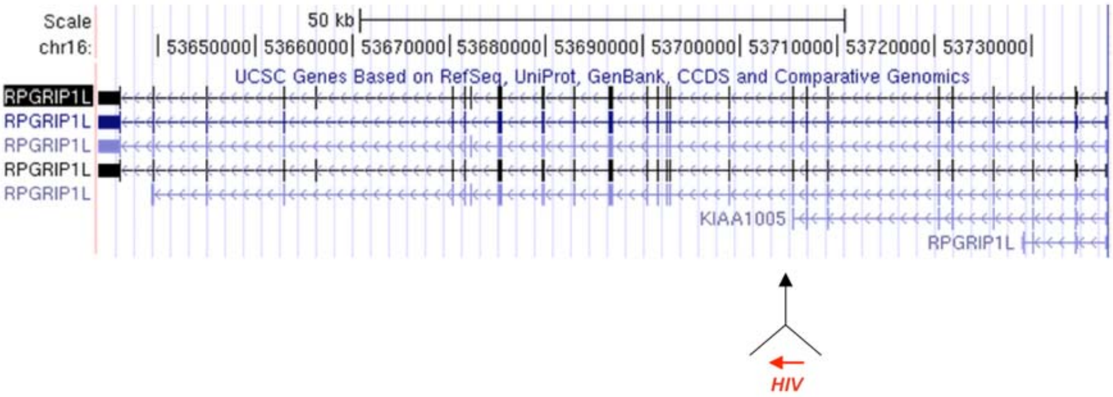


```
----------------------------------------------------------------------
chr16: 53709613-53709902
53709613   ATATGTTGCAGATGTGCCTTTAGCTGTAAGAGATCACTTGATGTCCACAGGGTATATAGA
53709673   AACCTTATATGTTACAATACTATTTTGTATTTTTCCTACTGAGATTAAAGCAAATGTAGA
53709773   ATTTAACAGGAAGATCTTGTTAAAG <--HIV-- GTTAATCATAGATCTAAATGAGTAC
53709783   CTGTTTTCAAAAGGCATTTATTTTCTTGACAGAGAAAAGTTCCTTAATTTAATACATTTT
53709843   ATATTAAAAAATATTTTGTTAATTTTGAAGCTTCATTTCAGAGTTCACTTTTAGGAAAAA
----------------------------------------------------------------------
```

Figure S4. Integration site on chr5q23.3. The integration site on chromosome 5, q23.3 mapped by high throughput sequencing of nrLAM-PCR products. The site is located in the fifth intron of the FBN2 (fibrillin 2, Gene ID: 2201) gene, co-linear with the direction of transcription (upper panel). The lower panel shows the genomic sequences flanking the integration site, with nucleotides covered by the longest read (chr5: 127,801,421-127,801,706) highlighted in blue.

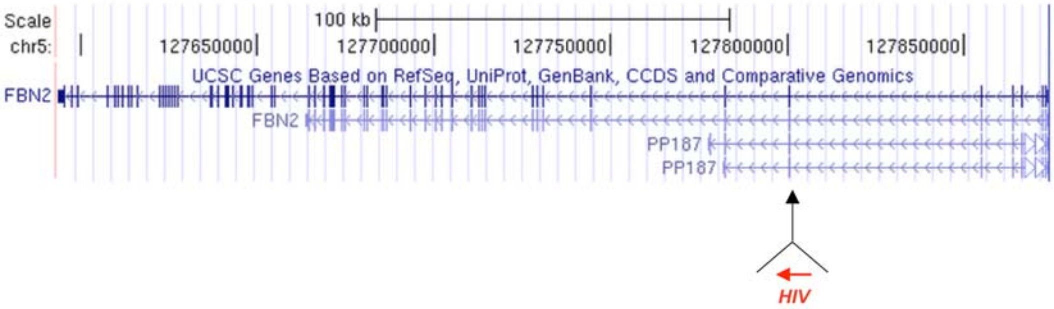


```
---------------------------------------------------------------------
chr5:127801276-127801685
127801276 TCAGTCAGTTTAGTTCTATGGGTAGCCTAGTTAGGAAGATCTCATTAATAAGGCACATAG
127801336 TCATGAATTCCTTACACGTAAGTTAGTATTATATGATCGCTCCTCTCTAGCAAGTGACTG
127801396 TACATTGTCCAGCTCTGTTATGGTG <--HIV-- ATAACAAAAGCCTTAGTAAATGTAT
127801446 GGCATGTGTGATACATAGGCAAATTCTTCTATGAATATTAAGTACAGTCCATTTAGATCC
127801506 CCGCCTCTCAAGGTTTCTATGGACCAGCAGCAACAGCATCACTTTGGGGCCGGGTAGAAA
127801566 TGCAGACTCAAGCCCTACCCTGACCCACTAACCCAGGTTCTGCATTTTTTAACAAGACCG
127801626 GGCATTAATATGCACCTTGGAGTTTCAGAAGGGCTGATATAGACTGCAGGCAGTTGGAAA
---------------------------------------------------------------------
```

Figure S5. Integration site on chr11q13.2. The integration site on chromosome 11, q13.2 mapped by high throughput sequencing of nrLAM-PCR products. The site is located in the first intron of the PACS (phosphofurin acidic cluster sorting protein 1, Gene ID: 55690) gene, co-linear with the direction of transcription (upper panel). The lower panel shows the genomic sequences flanking the integration site, with nucleotides covered by the longest read (chr11: 65,947,821-65,948,072) highlighted in blue.

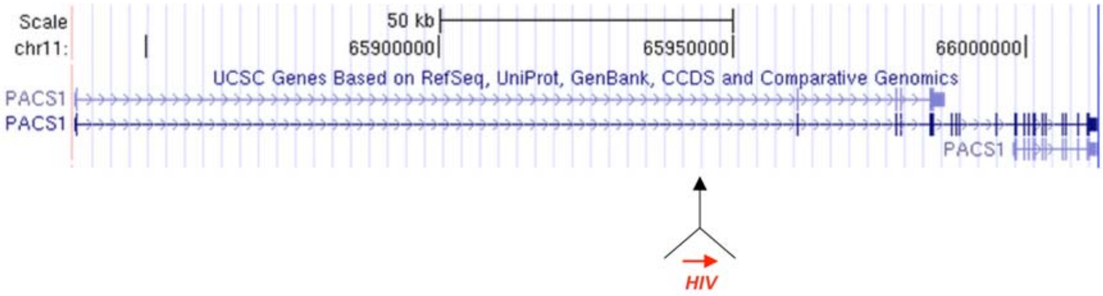


```
--------------------------------------------------------------------- chr1
1:65947748-65948217
65947748   GTTGATTCTCTTGAGATCCAATAATACACAGTCATACATTTACAAATAAGGATTGTATTG
65947808   TCTCTTTCTTTCAGCTTTTTTTTTTGTGTGTGTGTGTGAAACGGAGTCTCACTCTGTTGC
65947868   CAGGCTGGAGTGCAGTGGCGTGATCTCAGCTCACTGCAACCTCCCACTCCCTGATTCAAG
65947928   CAATTCTCCTGCCTCAGCTTCCTGAGTAGCTGGGATTACAGGCACGCACCACCACGCCTA
65947988   GCTAATTTTTGTATTTTTAGTAGAGACAGGGTTTCACCATGTTGGCCAAAATGGTCTCGA
65948048   TCTCCTGACCTCATGATCCGCCTAC  --HIV-- CTCAGCCTCCCAAAGTGCTGGAATT
65948098   ACAGGCGTGCGCCACCGCACCCAGCCTTATTTTTTTACTTACTTAATTTTGAGATAGGAT
65948158   GTTGCTCTCTCAACCAGGCTGGATTGCAGTGGTATGATCATAGCCCACTGCAGCCTTGAC
---------------------------------------------------------------------
```

Figure S6. Cellular growth curves upon Tre expression. Exponentially growing LV-cCtr or LV-cTre (constitutive promoter configuration) transduced Jurkat T cells, or mock-transduced cells, were seeded into separate wells, expanded and split as required. Cell numbers were counted at the indicated days after seeding (corresponding to week 13 to 15 of constitutive Tre expression).

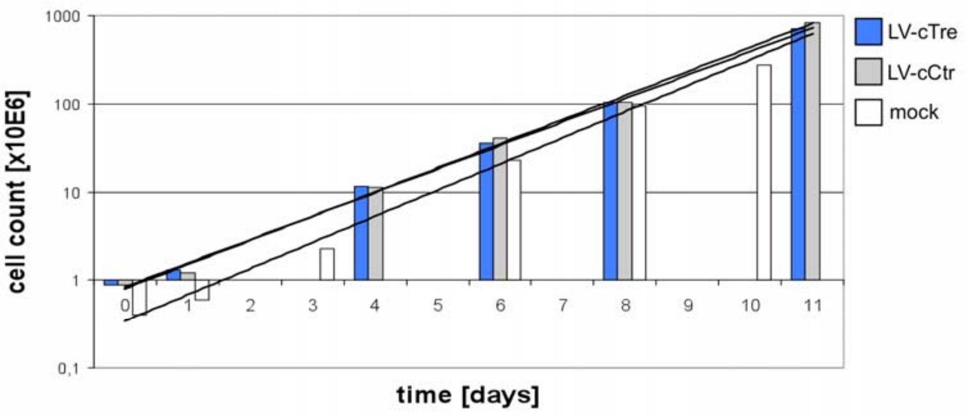

Figure S7. Array-CGH analysis of DNA isolated from primary human CD4⁺ T cells overexpressing Tre compared to mock-transfected cells. Numbers at the top of every panel indicate the respective chromosome (for chromosome 17 see Figure 4B). Normal log2 ratios of color intensities (-4 to +4) for each probe populate the charts. Heterozygous deletions would be indicated by green dots with a value around -1. Heterozygous duplications would be indicated by red dots with a value around 0.585.

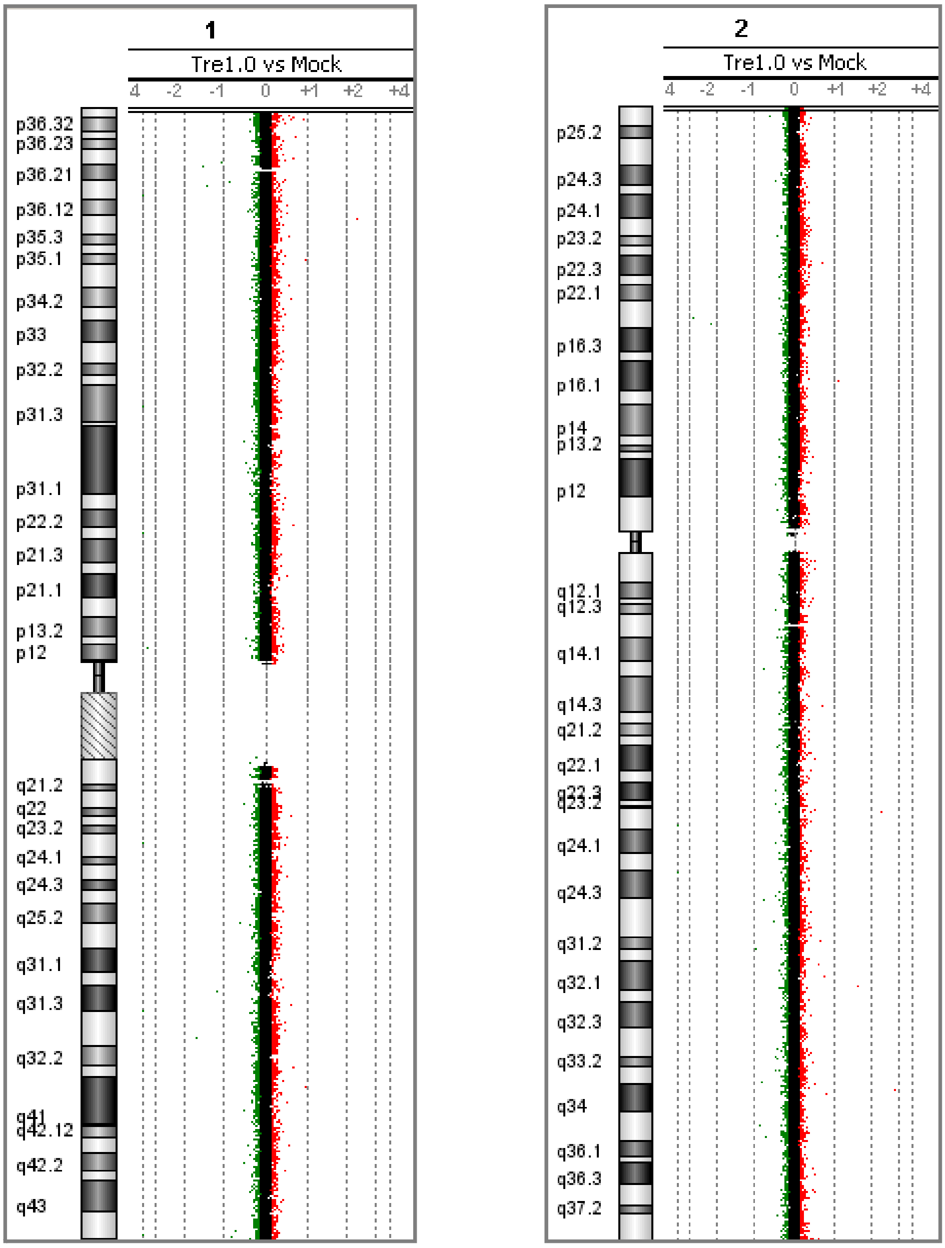

Figure S7. continued

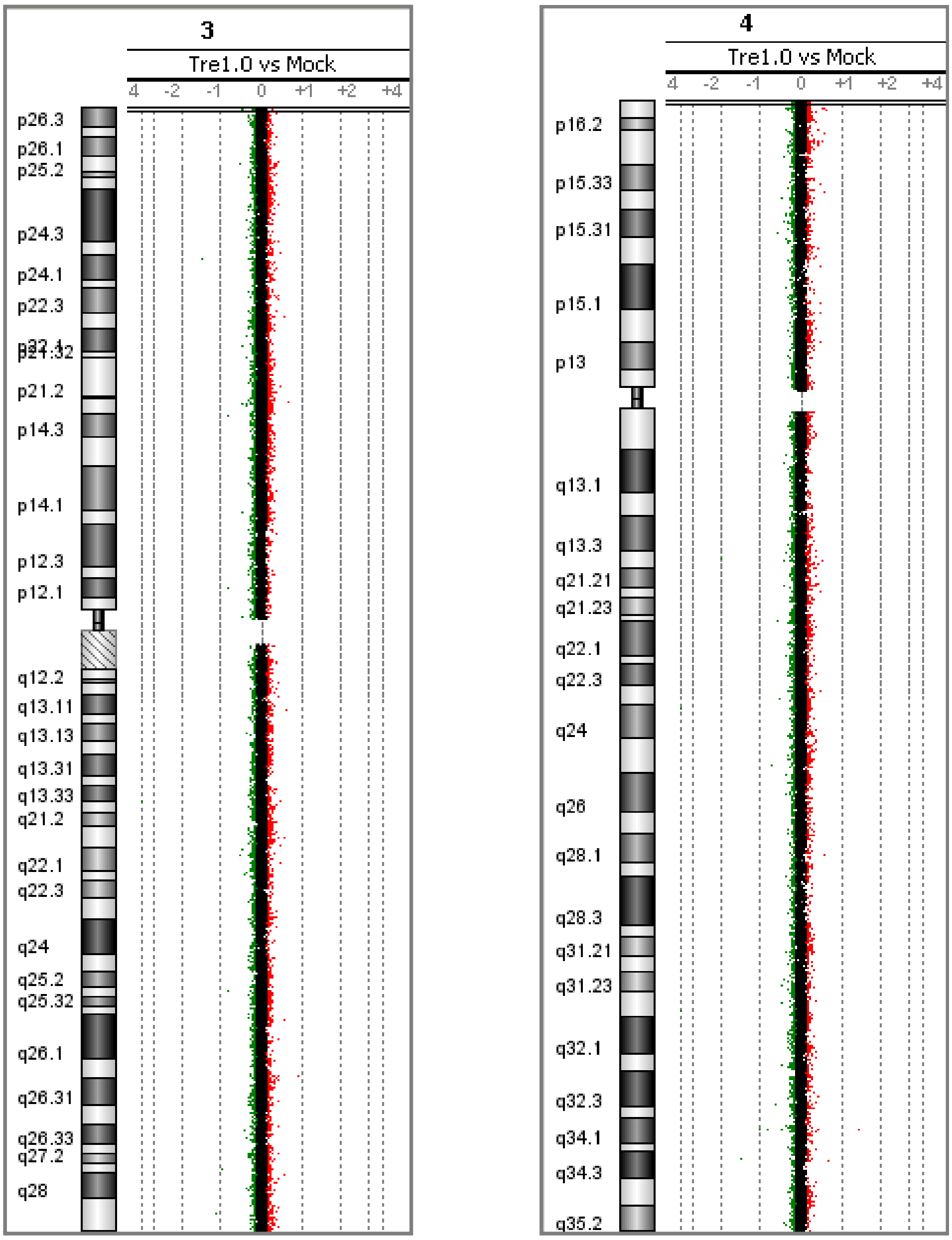

Figure S7. continued

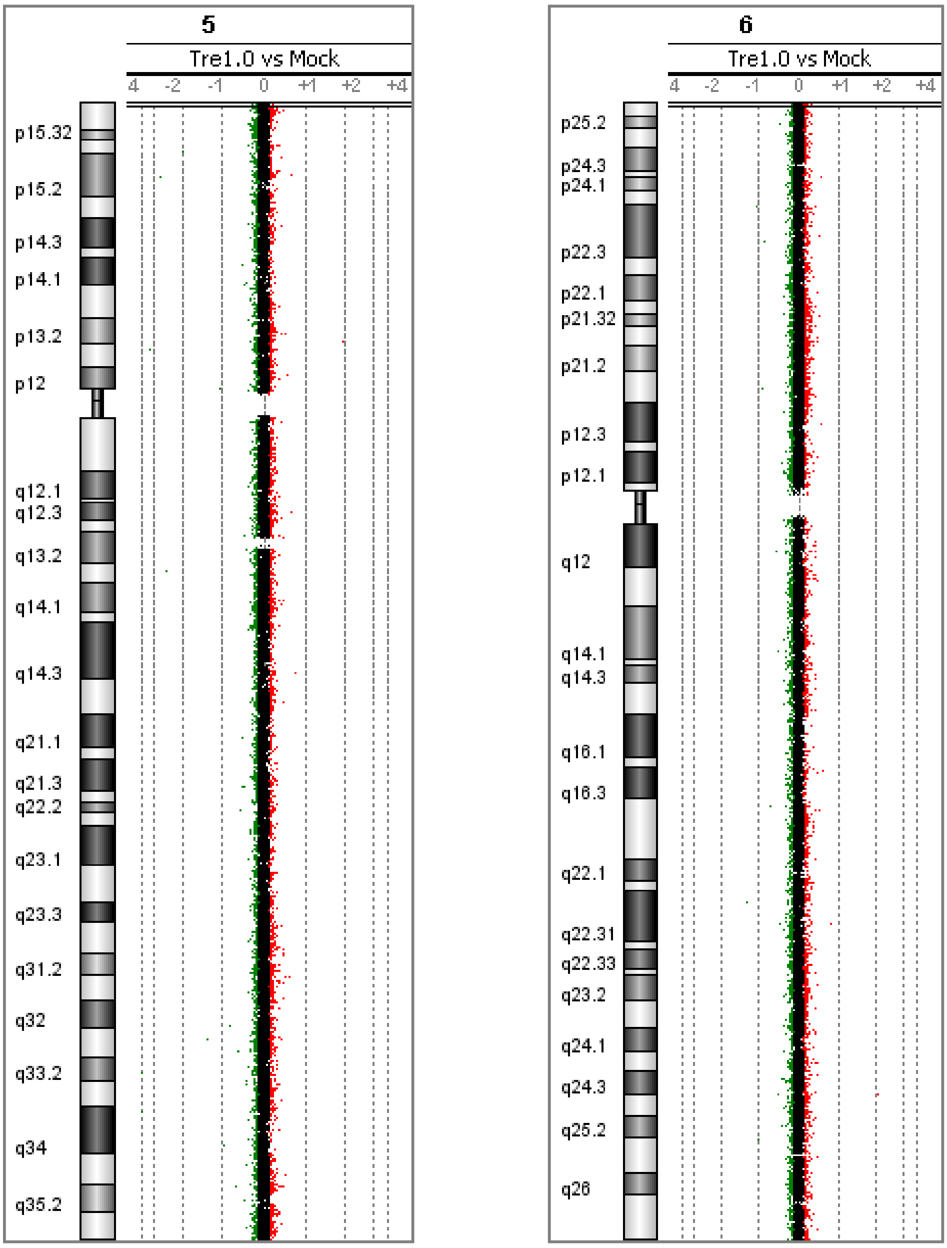

Figure S7. continued

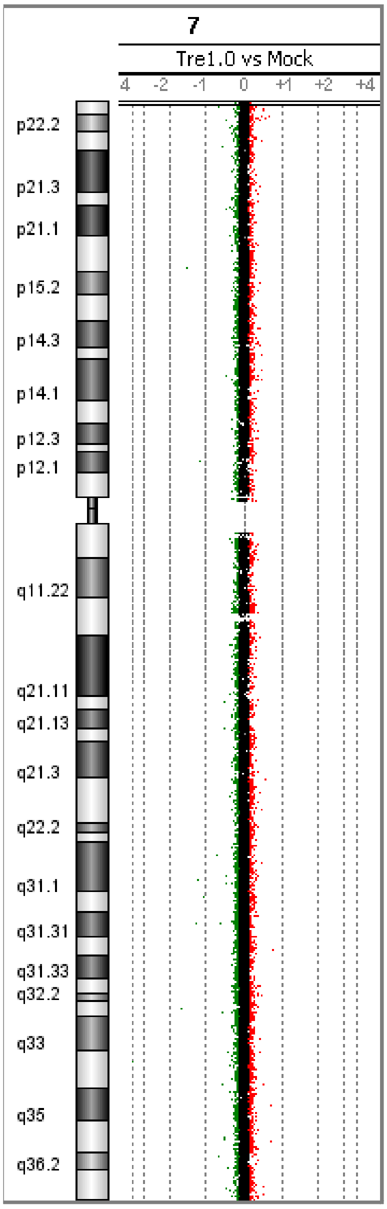

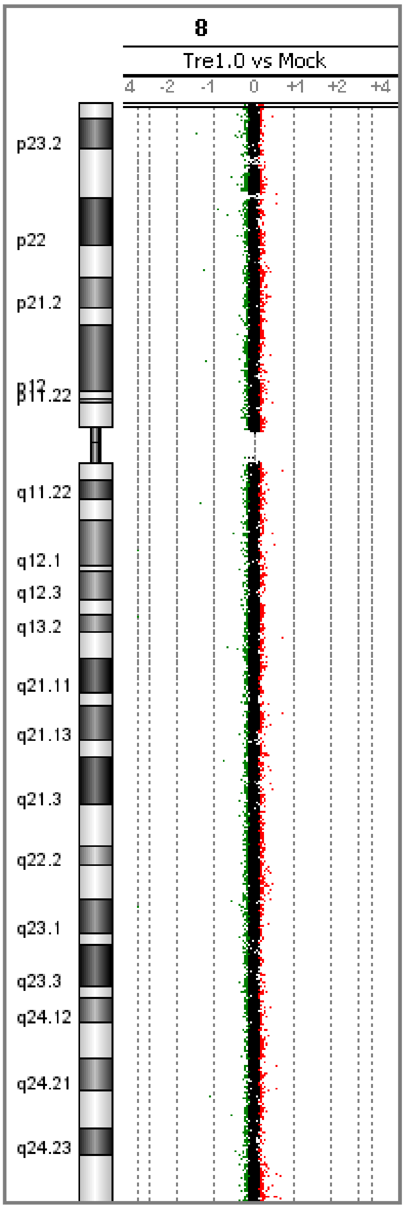

Figure S7. continued

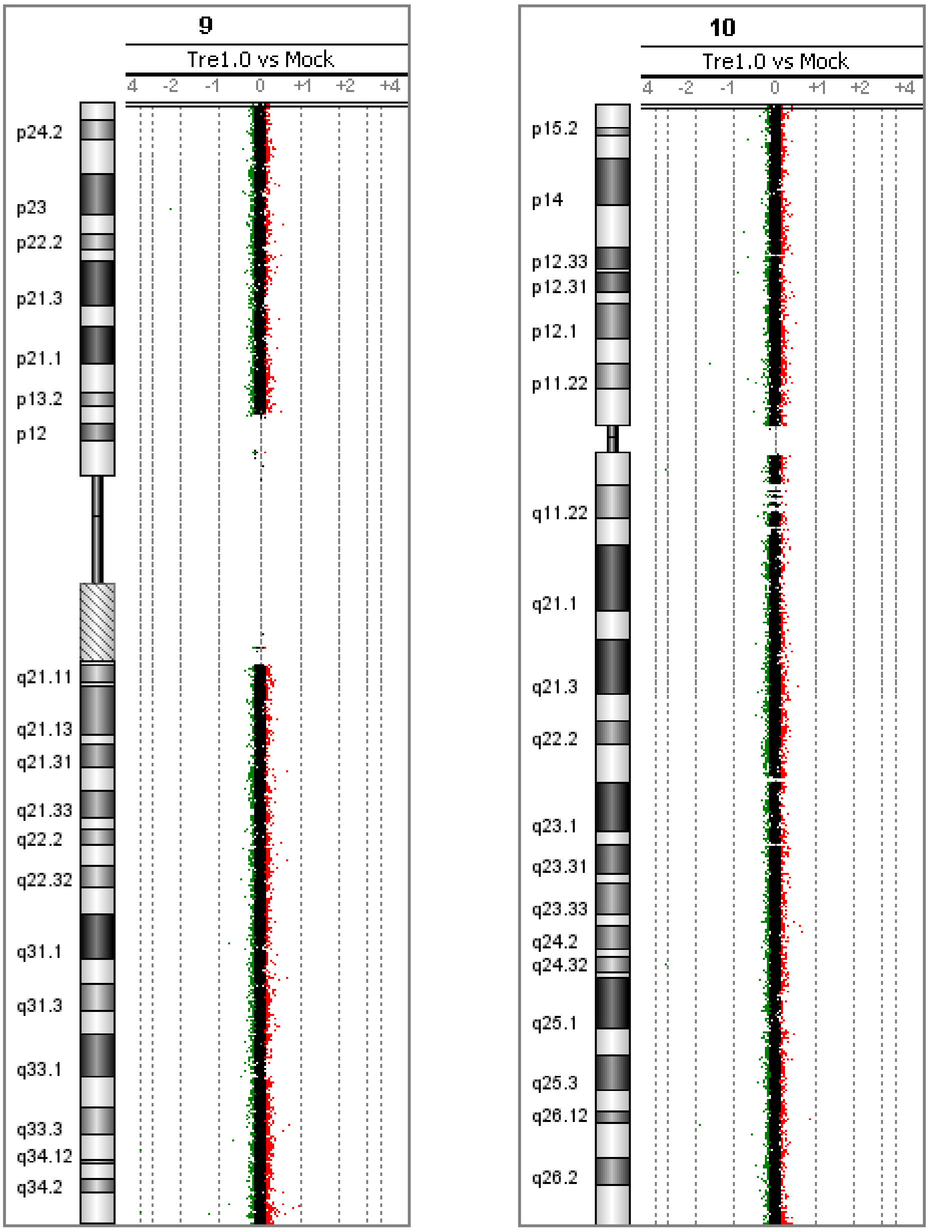

Figure S7. continued

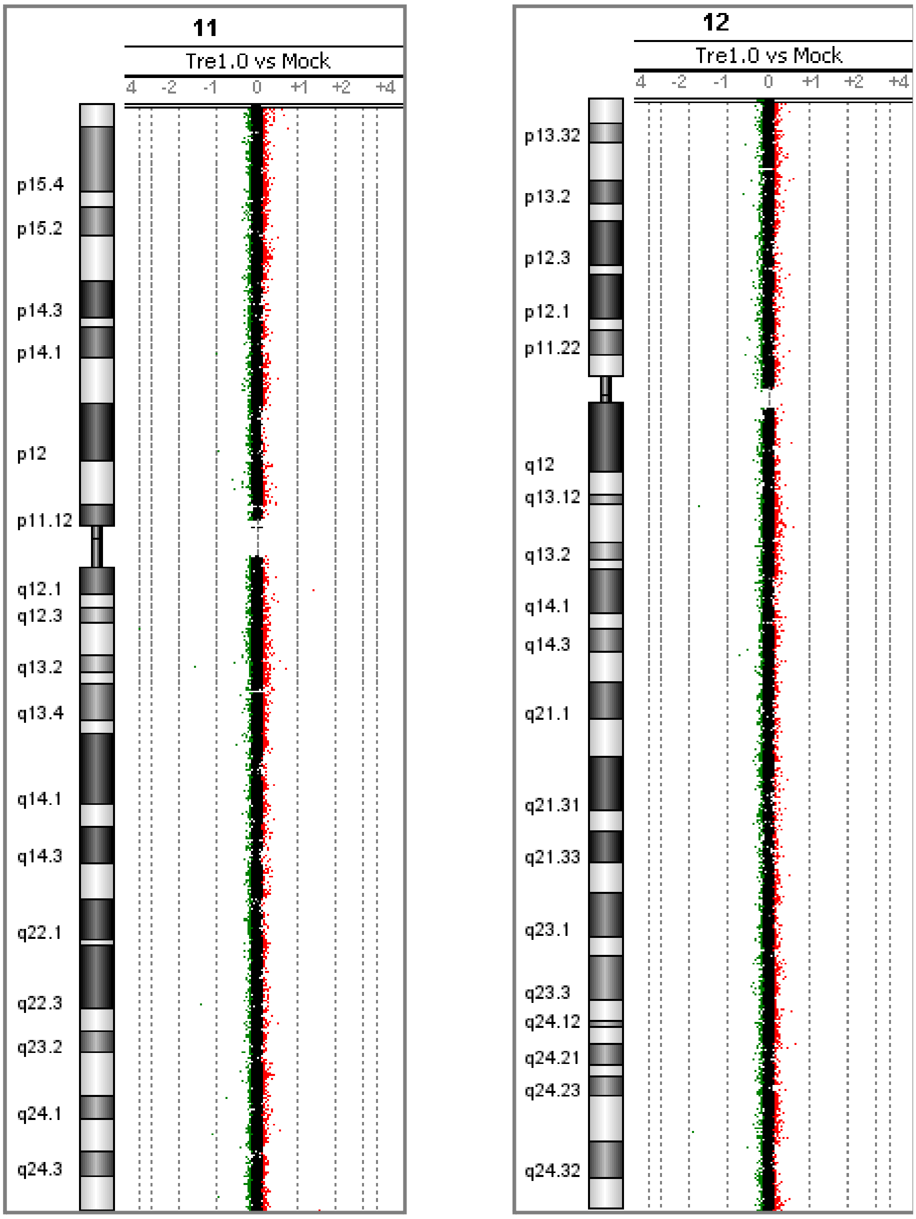

Figure S7. continued

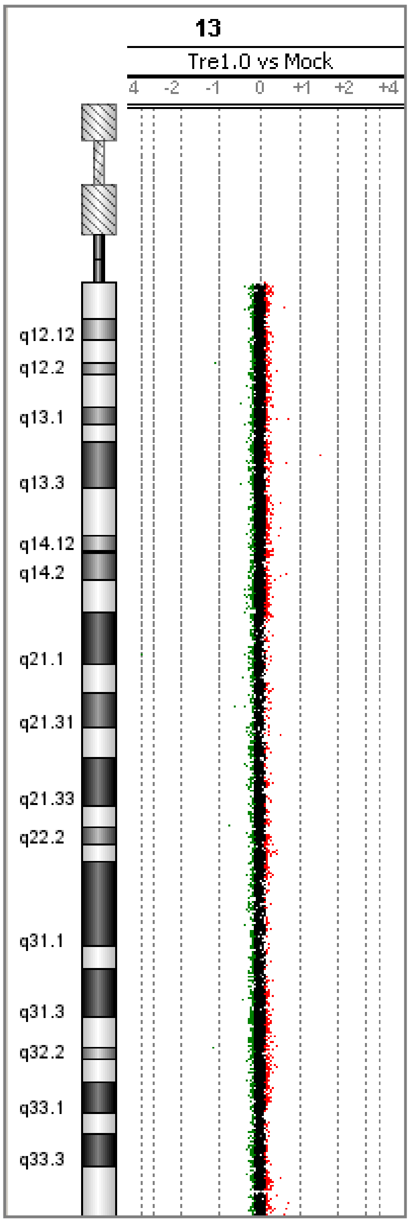

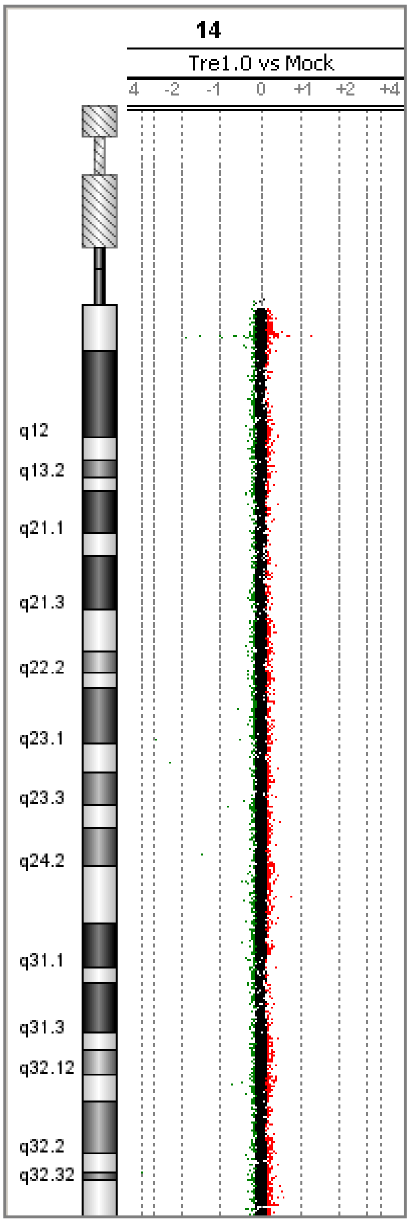

Figure S7. continued

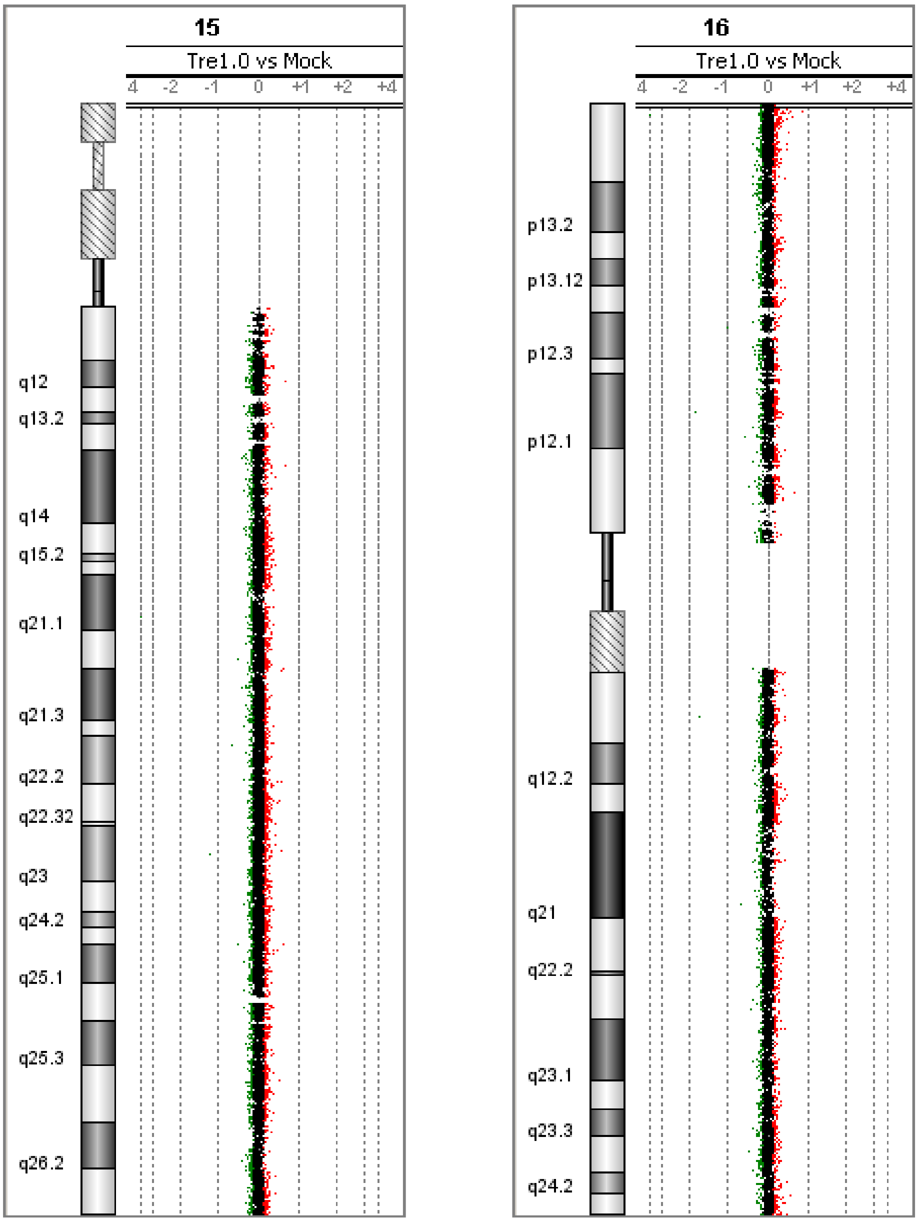

Figure S7. continued

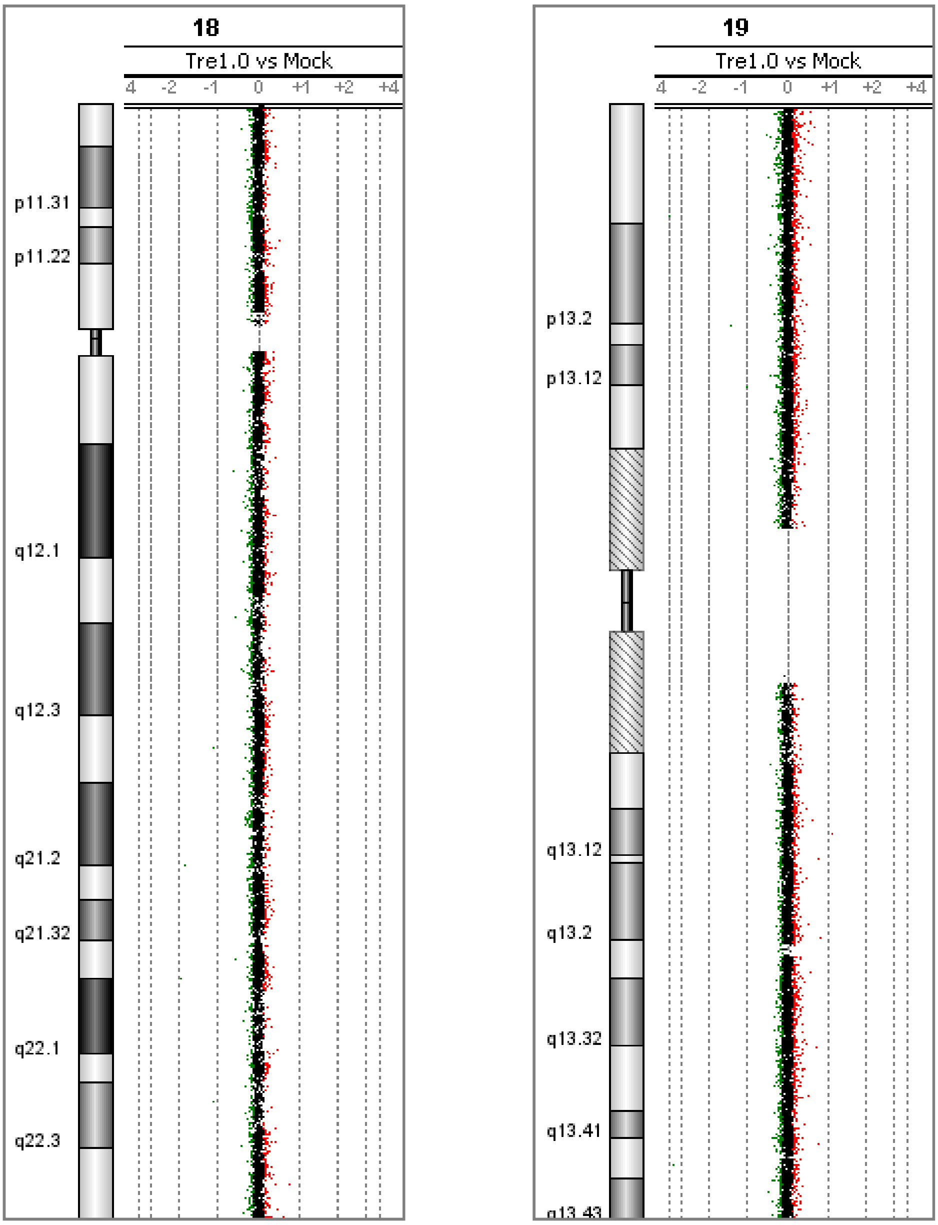

Figure S7. continued

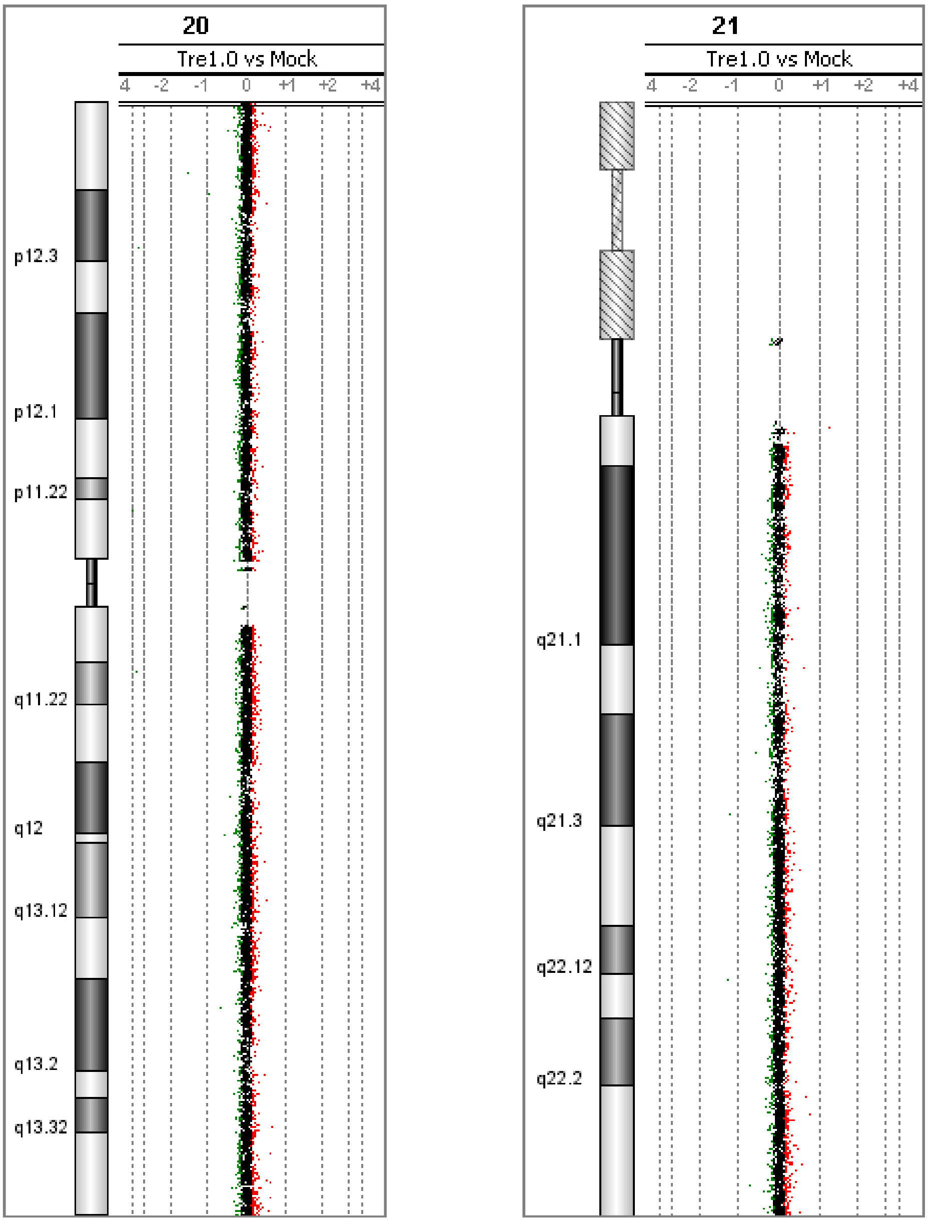

Figure S7. continued

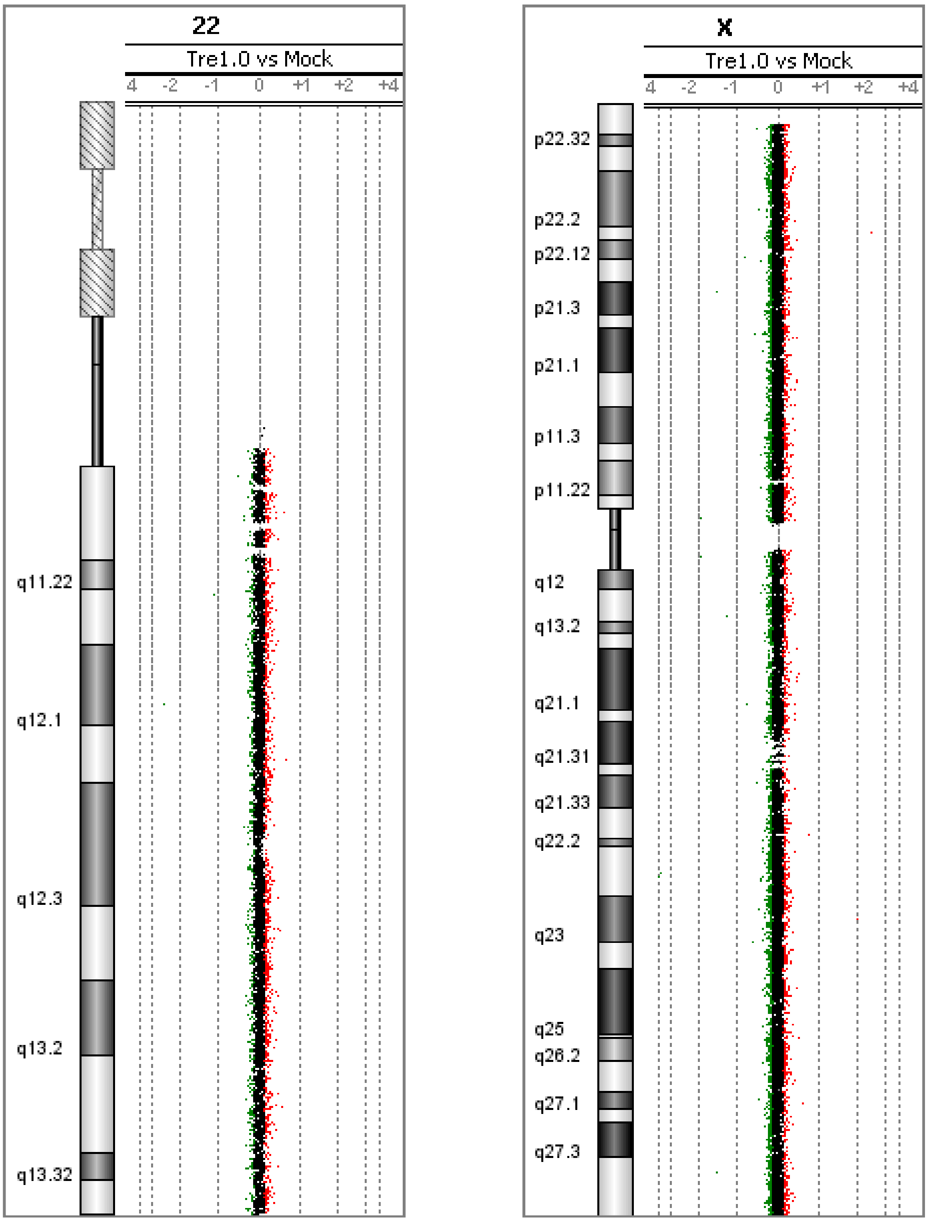

Figure S8. Tre activity against different HIV-1 isolates. (A) Tre-recombinase was raised against the Tre target site (loxLTR) in the primary HIV-1 strain TZB0003. The respective loxLTR sites in the independent clinical isolates TZB0065/03RP and 03SP100655 are characterized by a single nucleotide mismatch (indicated in red). (B) Agarose gel showing the activity of Tre on loxLTR (lanes 1-2) and on loxLTR-like sites present in the different HIV-1 strains (lanes 3-6), respectively. BsrG I / Xba I restriction digest results in a 5.2 kb fragment for non-recombined plasmid (two triangles) and a 4.2 kb fragment for recombined product (one triangle). -, non-induced; +, induced with 1 mg/ml L-arabinose; M, DNA marker lane.

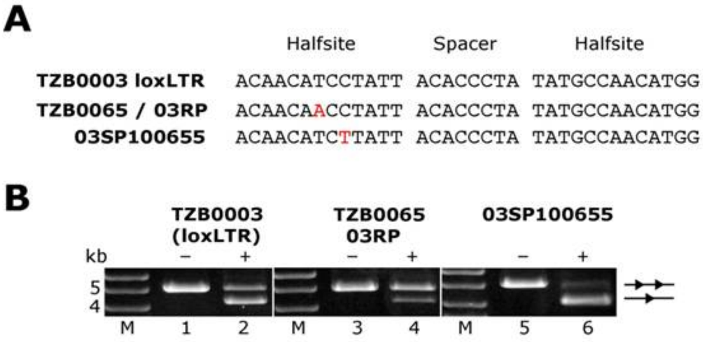

Figure S9. Analysis of HIV-1 coreceptor expression on LV-transduced human peripheral blood CD4⁺ T cells. (A) An unselected LV-Tre transduced CD4⁺ T cell pool was analyzed by flow cytometry (left panels). The majority of CD4⁺ T cells expressed the CCR5 surface receptor (mean: 30.3%), as compared to the CXCR4 molecule (mean: <2%; right panels). (B) Flow cytometric analysis of control vector (LV-Ctr) transduced CD4⁺ T cells (left panels). CCR5⁺ T cells (mean 14.8%) and CXCR4⁺ T cells (mean 0.17%) are shown.

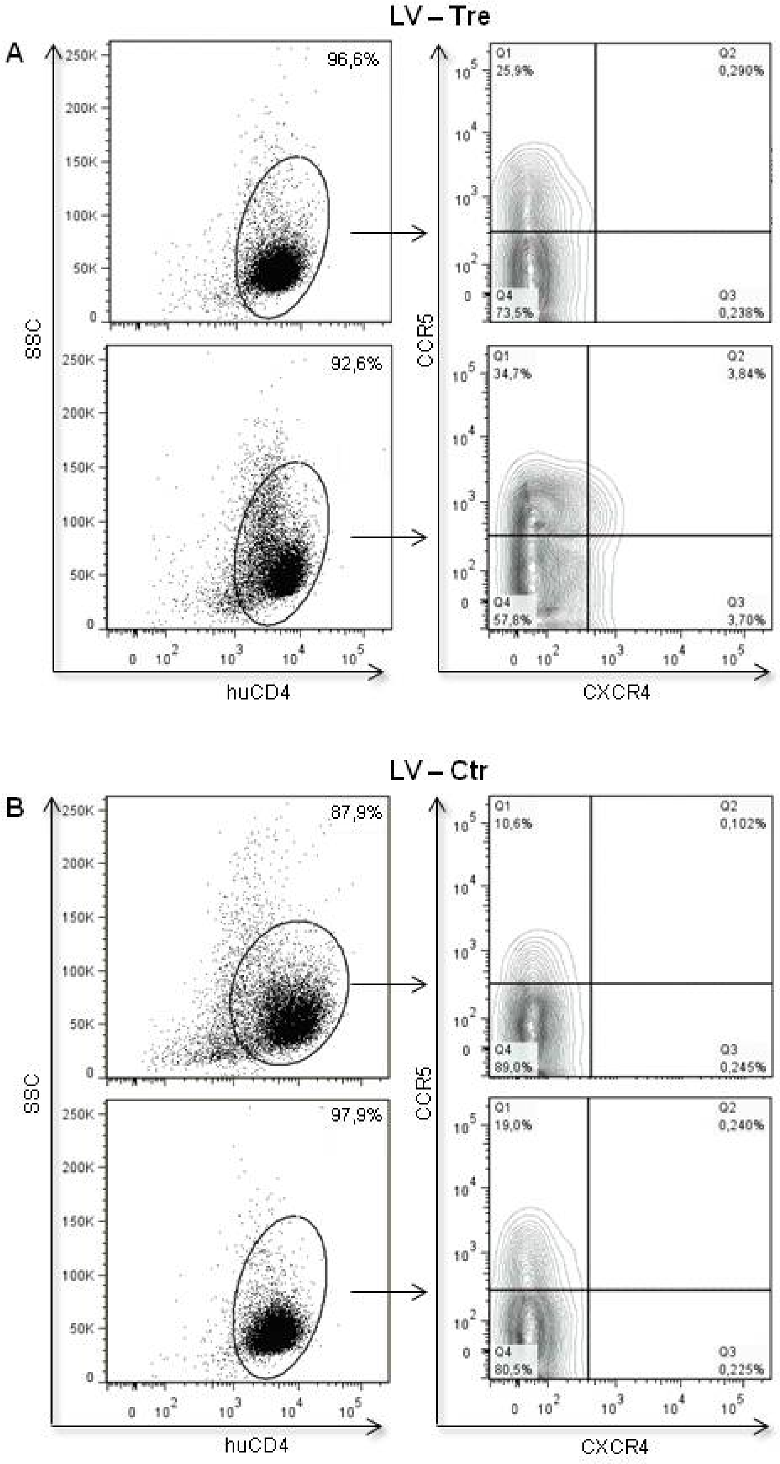

Figure S10. FACS analysis of single cell suspensions derived from various organs. At sixteen weeks post HIV-1 infection, cells derived from organs of representative euthanized mice, transplanted with LV-Tre (left panels) or LV-Ctr (right panels) transduced CD4^{+} T cells, were analyzed by flow cytometry for the indicated marker proteins. Single cell suspensions were prepared from (A) Bone Marrow (BM); (B) Liver; (C) Spleen; (D) Thymus.

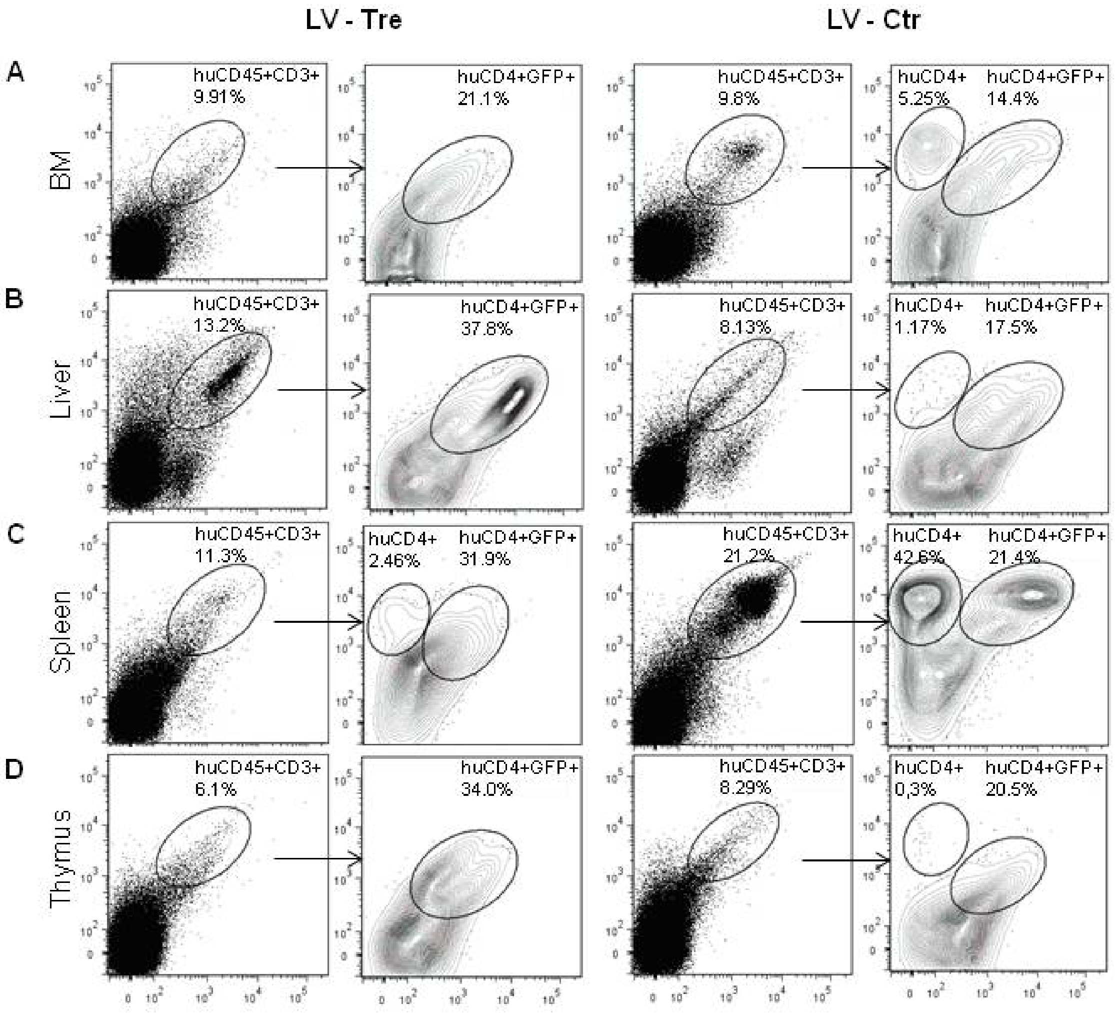

Figure S11. FACS analysis of single cell suspensions derived from various organs of HIV-infected mice transplanted with LV-Tre transduced CD34[+] HSC. At twelve weeks post HIV-1 infection, cells derived from (A) Bone Marrow or (B) Spleen of representative euthanized mice were analyzed by flow cytometry for the indicated marker proteins.

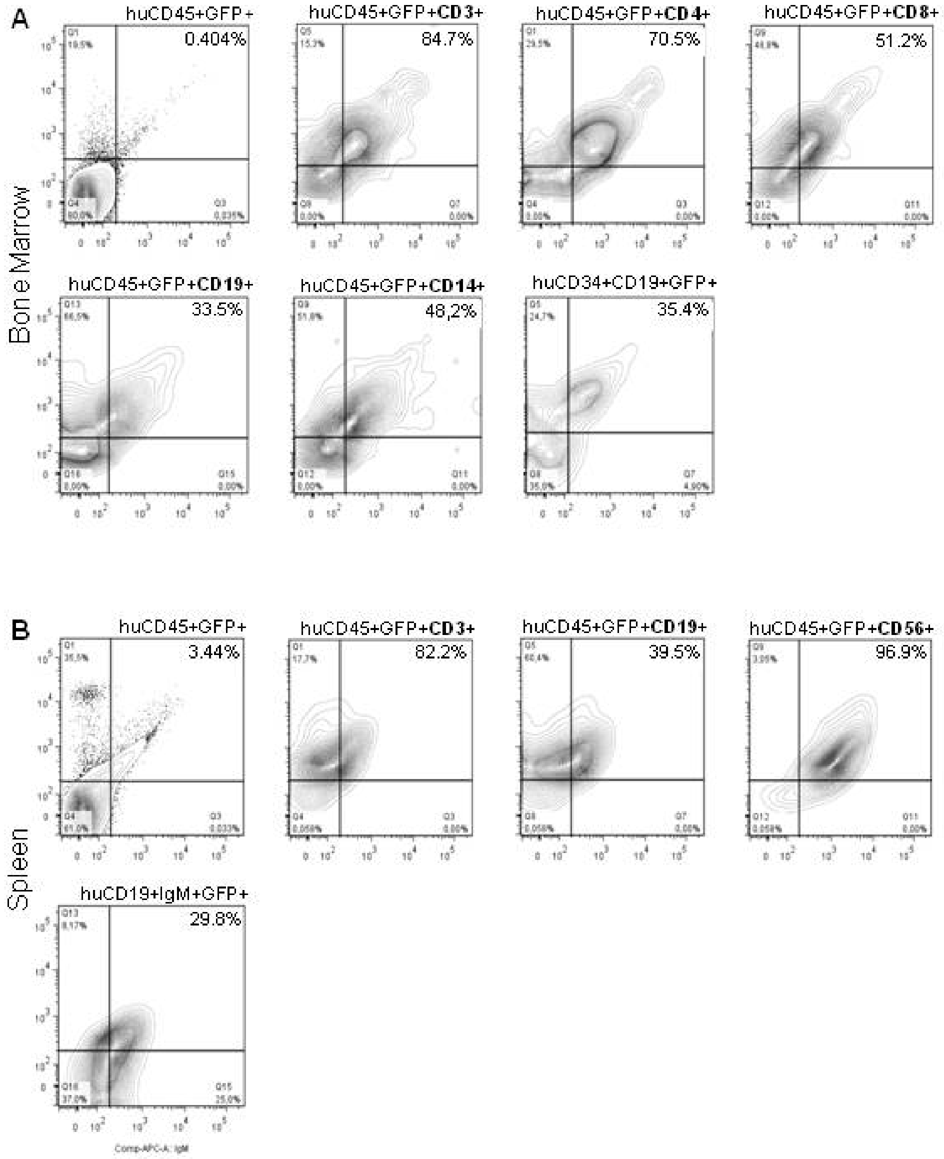

Table S1.

Sequences of bar-coded fusion primers used for pyrosequencing.

**Name**	**Sequence** [A]
LVTre-A	5'- *CGTATCGCCTCCCTCGCGCCATCAG***ACGCTCGACA**AGGGAAG TAGCCTTGTGTGTG -3'
LVTre-B	5'- *CTATGCGCCTTGCCAGCCCGCTCAG***CTCGCGTGTC**GATCTGAA TTCAGTGGCACAG -3'
LVCtr-A	5'- *CGTATCGCCTCCCTCGCGCCATCAG***TCACGTACTA**AGGGAAGT AGCCTTGTGTGTG -3'
LVCtr-B	5' – *CTATGCGCCTTGCCAGCCCGCTCAG***TACGAGTATG**GATCTGAA TTCAGTGGCACAG -3'

[A] Multiplex identifier sequences are shown in bold face, fused GS FLX-specific adaptors A or B are shown in italics, and sequences complementary to the HIV-LTR (LVTre-A, LVCtr-A) or the nrLAM-PCR adaptor (LVTre-B, LVCtr-B) are underlined. Sequencing was performed using the fusion adaptor A.
